# Cell Type-Specific Oxidative Stress Genomic Signatures in the Globus Pallidus of Dopamine-Depleted Mice

**DOI:** 10.1523/JNEUROSCI.1634-20.2020

**Published:** 2020-12-09

**Authors:** Alyssa J. Lawler, Ashley R. Brown, Rachel S. Bouchard, Noelle Toong, Yeonju Kim, Nitinram Velraj, Grant Fox, Michael Kleyman, Byungsoo Kang, Aryn H. Gittis, Andreas R. Pfenning

**Affiliations:** ^1^Computational Biology; ^2^Biological Sciences; ^3^Neuroscience Institute, Carnegie Mellon University, Pittsburgh, Pennsylvania 15213

**Keywords:** epigenomics, globus pallidus, Hif2a, oxidative stress, Parkinson's disease, parvalbumin

## Abstract

Neuron subtype dysfunction is a key contributor to neurologic disease circuits, but identifying associated gene regulatory pathways is complicated by the molecular complexity of the brain. For example, parvalbumin-expressing (PV^+^) neurons in the external globus pallidus (GPe) are critically involved in the motor deficits of dopamine-depleted mouse models of Parkinson's disease, where cell type-specific optogenetic stimulation of PV^+^ neurons over other neuron populations rescues locomotion. Despite the distinct roles these cell types play in the neural circuit, the molecular correlates remain unknown because of the difficulty of isolating rare neuron subtypes. To address this issue, we developed a new viral affinity purification strategy, Cre-Specific Nuclear Anchored Independent Labeling, to isolate Cre recombinase-expressing (Cre^+^) nuclei from the adult mouse brain. Applying this technology, we performed targeted assessments of the cell type-specific transcriptomic and epigenetic effects of dopamine depletion on PV^+^ and PV^–^ cells within three brain regions of male and female mice: GPe, striatum, and cortex. We found GPe PV^+^ neuron-specific gene expression changes that suggested increased hypoxia-inducible factor 2α signaling. Consistent with transcriptomic data, regions of open chromatin affected by dopamine depletion within GPe PV^+^ neurons were enriched for hypoxia-inducible factor family binding motifs. The gene expression and epigenomic experiments performed on PV^+^ neurons isolated by Cre-Specific Nuclear Anchored Independent Labeling identified a transcriptional regulatory network mediated by the neuroprotective factor Hif2a as underlying neural circuit differences in response to dopamine depletion.

**SIGNIFICANCE STATEMENT** Cre-Specific Nuclear Anchored Independent Labeling is an enhanced, virus-based approach to isolate nuclei of a specific cell type for transcriptome and epigenome interrogation that decreases dependency on transgenic animals. Applying this technology to GPe parvalbumin-expressing neurons in a mouse model of Parkinson's disease, we discovered evidence for an upregulation of the oxygen homeostasis maintaining pathway involving Hypoxia-inducible factor 2α. These results provide new insight into how neuron subtypes outside the substantia nigra pars compacta may be compensating at a molecular level for differences in the motor production neural circuit during the progression of Parkinson's disease. Furthermore, they emphasize the utility of cell type-specific technologies, such as Cre-Specific Nuclear Anchored Independent Labeling, for isolated assessment of specific neuron subtypes in complex systems.

## Introduction

A unique challenge for identifying pathologic gene regulation in neurologic diseases is the extreme heterogeneity of brain tissues, which are often comprised of dozens of molecularly distinct cell types and neuron subtypes (Lake et al., 2018; Saunders et al., 2018; Tasic, 2018). Even abnormal expression of a single gene within a minority cell population can exert outsized effects on the system (Wen et al., 2010; Dutton et al., 2013). One neuron subtype important in many diseases is fast-spiking parvalbumin-expressing (PV^+^) neurons, which are essential for maintaining balance between excitatory and inhibitory influences brainwide (Hu et al., 2014; Ferguson and Gao, 2018).

In the basal ganglia, PV^+^ neurons are optimally positioned to regulate the motor circuits involved in movement disorders, such as Parkinson's disease (PD). PV^+^ neurons constitute roughly half of all neurons in the GPe, a key contributor to pathogenic hypersynchrony of basal ganglia nuclei under the dopamine-depleted conditions of PD (Bergman et al., 1998; Bevan et al., 2002; Mallet et al., 2008). GPe PV^+^ neurons, also called prototypical neurons, have fast, regular firing properties and strongly project to the subthalamic nucleus, the major output nucleus for action (Hegeman et al., 2016; Saunders et al., 2016). Behaviorally, optogenetic stimulation of GPe PV^+^ neurons, but not global GPe stimulation, has prokinetic effects in mice with late-stage dopamine depletion (DD) (Mastro et al., 2017). Additionally, reduced numbers of GPe PV^+^ neurons have been reported in postmortem brain tissue of PD patients and certain animal models of PD (Hardman and Halliday, 1999; Fernández-Suárez et al., 2012).

Despite the associations between PV^+^ neurons and PD, the molecular correlates of disease in these and other neuron populations are understudied because of technical barriers. Bulk tissue RNA sequencing (RNA-seq) (Wang et al., 2009) and the assay for transposase-accessible chromatin (ATAC-seq) (Buenrostro et al., 2013, 2015) are ideal for identifying gene expression and chromatin landscapes in homogeneous tissues, and together provide a rich snapshot of gene regulation. With increased heterogeneity, though, the interpretability of bulk tissue assays breaks down because subpopulations exist in different transcriptomic and epigenetic states and experience independent changes.

In recent years, the need for molecular access to specific cell populations has triggered rapid technology development, but there are particular shortcomings pertinent to neuroepigenetics that have yet to be optimized. Manual dissection, laser capture microdissection, and FACS-based isolation technologies have all been applied toward transcriptomic profiling of isolated populations, including certain groups of PV^+^ neurons (Sugino et al., 2006; Pietersen et al., 2014; Enwright et al., 2018). Pairing these sorting techniques with ATAC-seq has been suitable for broad, abundant cell classes in the brain (Fullard et al., 2017; Nott et al., 2019), but remains challenging for small populations because of nuclei fragility and low yield (Handley et al., 2015; Mo et al., 2015). Given the rarity of neurons compared with glia in the GPe (von Bartheld et al., 2016), these sorting methods are unlikely to produce high-quality epigenomic data.

A more recent nuclei isolation approach with advantages in epigenomics compatibility is INTACT. This method, which induces cell type-specific expression of a nuclear surface tag, was first developed in plants (Deal and Henikoff, 2010) and later adapted for murine neuronal subtypes, including PV^+^ neurons (Mo et al., 2015). INTACT labeling can be used in combination with FACS (Chamessian et al., 2018) or with immunopurification, which is more gentle and effective for rare cell types (Handley et al., 2015; Mo et al., 2015). Relative to emerging single nucleus RNA-seq and ATAC-seq technologies, targeted profiling of specific labeled cell populations with INTACT is more efficient, especially if the population of interest is rare. Still, INTACT requires double-transgenic mice, which are time-consuming to maintain and may not be compatible with conditional disease models. To relieve some of this burden, we developed a viral INTACT system for isolating nuclei: Cre-Specific Nuclear Anchored Independent Labeling (cSNAIL). This technology combines the ubiquity of cell type-specific Cre transgenic animals with the convenience of adeno-associated viruses (AAVs). cSNAIL is particularly well suited for rare neuron subtype isolation, and the principles can scale beyond the means of transgenic technologies.

Here, we used cSNAIL to interrogate whether DD induces gene expression and chromatin accessibility changes in GPe PV^+^ neurons that might reflect or contribute to the pathology of PD. In parallel, we conducted RNA-seq and ATAC-seq on cSNAIL-isolated PV^+^ populations and the remaining PV^–^ cells from 6-OHDA-lesioned mice and sham controls. In order to determine the extent to which DD-induced gene regulatory effects in GPe PV^+^ neurons were region- and cell type-specific, we also assessed PV^+^ neurons and PV^–^ cells in two additional regions of differing proximities to GPe, the striatum and cortex. With this strategy, we identified GPe PV^+^ neuron-specific epigenetic and transcriptional responses to DD that were related to cellular oxygen sensing and oxidative stress, providing new insight into the molecular consequences of DD.

## Materials and Methods

### 

#### 

##### Experimental design

The main experiments were designed to assess gene expression and chromatin accessibility signatures in cSNAIL-isolated cell populations from mice with normal and impaired dopamine production. A cohort of male littermates (*N* = 3) and a cohort of female littermates (*N* = 3), all adult heterozygous *Pvalb-2A-Cre* mice, were transduced with cSNAIL AAV to label PV^+^ neurons. After 2-3 weeks of incubation, the animals underwent surgery for either a 6-OHDA injection to induce DD (2 males, 2 females) or a comparable saline injection (1 male, 1 female). This control allowed us to minimize possible confounding effects from the surgical procedure and isolate the effects of dopamine depletion itself. Three to five days after surgery, tissue from the cortex, striatum, and GPe was collected and sorted into labeled (PV^+^) nuclei and unlabeled nuclei via affinity purification with cSNAIL. The six nuclei populations from each animal were then divided for ATAC-seq or RNA-seq. For more metadata information, see Extended Data Figure 2-1. Supporting experiments included image analysis of cSNAIL expression and antibody staining of a candidate protein involved in the response to DD.

##### AAV design

**Figure 1.**
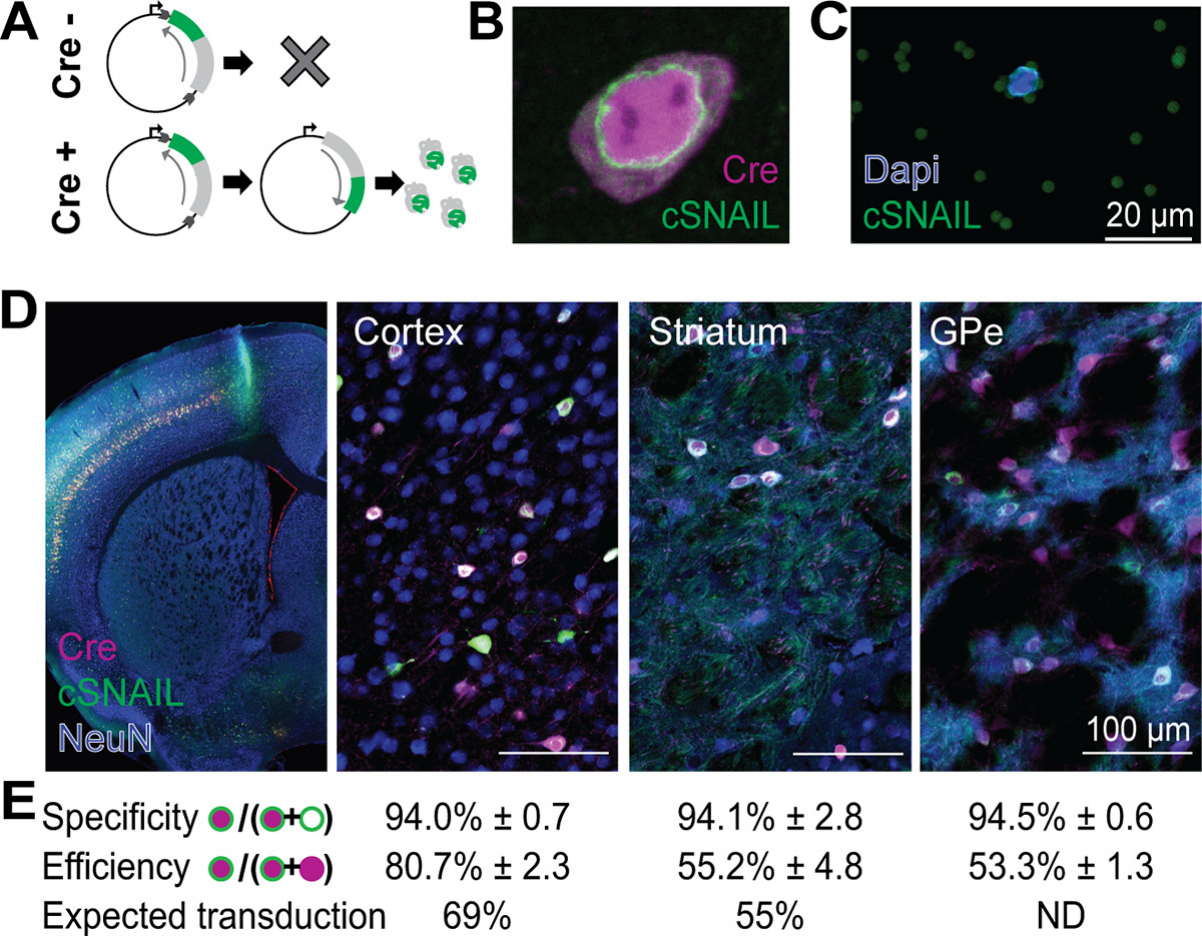
cSNAIL targets Cre^+^ nuclei with high precision. ***A***, Schematic for the Cre-dependent mechanism of Sun1GFP expression from cSNAIL. ***B***, Sun1GFP expression from cSNAIL correctly localized to the nuclear envelope in tissue. Shown is Sun1GFP from cSNAIL (green) in a Cre^+^ cell in cortical tissue from a *Pvalb-2A-Cre/Ai14* mouse, which expresses tdTomato as a reporter of Cre (magenta). ***C***, Sun1GFP expression from cSNAIL was sufficient to bind anti-GFP-coated magnetic beads for affinity purification. ***D***, Sun1GFP expression from cSNAIL was restricted to Cre^+^ cells in the cortex, striatum, and GPe. These fluorescent images contain Sun1GFP expression from cSNAIL in a *Pvalb-2A-Cre/Ai14* mouse, and the tissue slices were also stained for NeuN, a pan-neuronal marker (in blue). ***E***, cSNAIL virus specificity and efficiency within each brain region. Standard errors were calculated across four independent images. The total Sun1GFP^+^ and/or Cre^+^ cells in the analysis were cortex (*N* = 1088 cells), striatum (*N* = 107 cells), and GPe (*N* = 773 cells). Expected neuron transduction metrics were reported by Chan et al. (2017). ND, No data.

Several modifications were made to the INTACT system to allow it to be delivered using AAV. (1) We used an N-truncated version of the *Mus musculus Sun1* gene (Sad1 and UNC84 domain-containing 1, transcript variant 5, NCBI Reference Sequence: NM_001256118.1), amino acids 208-757, that retains the ability to localize to the nuclear envelope in a manner that is resistant to detergent tissue homogenization and is similar to the variant described by Haque et al. (2010). (2) We incorporated only the DNA sequence of exonic regions of *Sun1*. (3) The *Sun1GFP* fusion gene included one copy of superfolder GFP instead of two. (4) We adjusted the Cre-dependent mechanism from a loxp-flanked stop codon to the double-inverted orientation (DIO) system. In the initial cSNAIL genome, the *Sun1GFP* gene is in the reverse orientation with respect to the promoter such that the gene cannot be expressed. When the virus encounters Cre recombinase protein (Cre), it acts on the double-flanking lox sites to excise the *Sun1GFP* gene and reinsert it in the correct orientation for transcription (see Fig. 1*A*).

**Figure 2.**
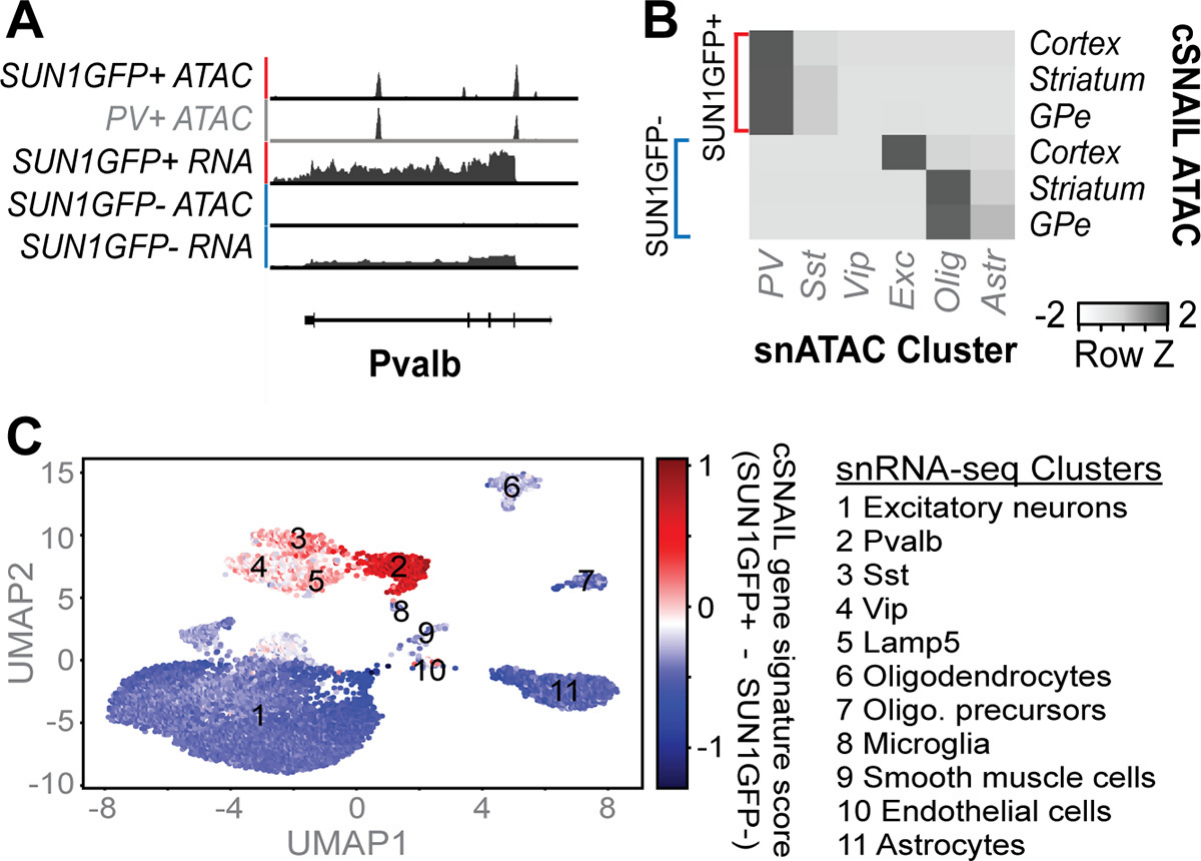
cSNAIL-isolated nuclei from *Pvalb-2A-Cre* mice recapitulate PV^+^ transcriptomic and epigenomic signals from external data. ***A***, Genome browser visualization of ATAC-seq and RNA-seq signal at the *Pvalb* locus from pooled *p* value tracks. cSNAIL data are labeled in black and are compared with publicly available data (Mo et al., 2015) labeled in gray. For detailed sample metadata, see Extended Data Figure 2-1. ***B***, Enrichments of mouse cortical snATAC-seq cluster markers (log2FoldDifference > 0.25 and *p* < 0.05 relative to all other clusters) within cell type-specific ATAC-seq peaks in our data by the hypergeometric test (snATAC-seq data and method from Signac vignette; https://github.com/timoast/signac). ***C***, Expression of cortical cSNAIL PV^+^ and PV^–^ gene signatures per cell of external mouse cortex snRNA-seq (data from https://portal.nemoarchive.org/; see Materials and Methods). Cluster assignments and Pvalb expression per cell can be found in Extended Data Figure 2-2.

10.1523/JNEUROSCI.1634-20.2020.f2-1 Figure 2-1 **Sample metadata per animal.** Details about the mice, treatments, and tissue collection for molecular analyses are provided. Download Figure 2-1, XLSX file.

10.1523/JNEUROSCI.1634-20.2020.f2-2 Figure 2-2 **Additional properties of the mouse motor cortex snRNA-seq data. A.** Cluster annotations per cell of the snRNA-seq data with UMAP embedding. **B.** Pvalb gene expression is highest within the Pvalb interneuron cluster. Download Figure 2-2, TIF file.

**Figure 3.**
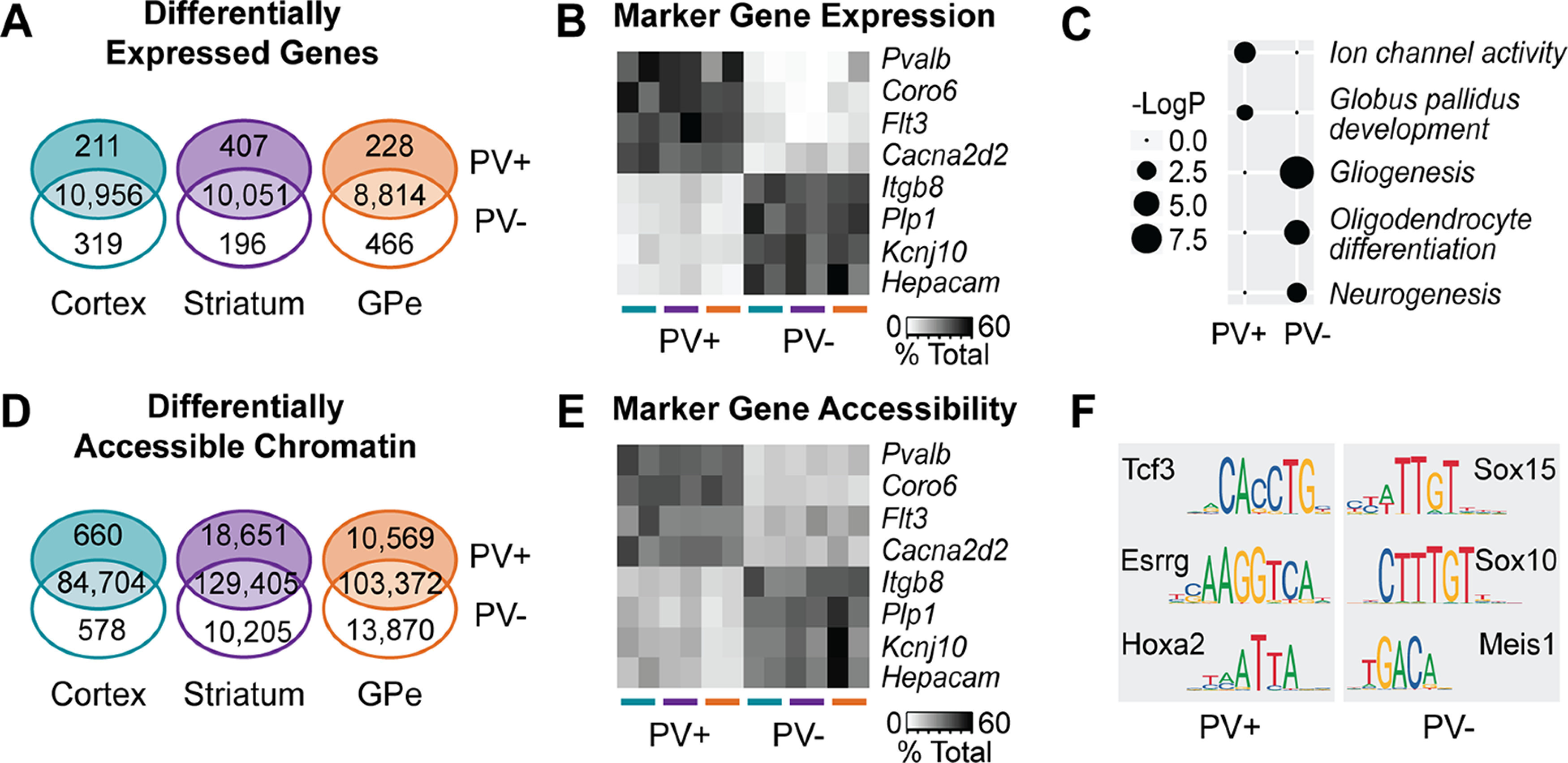
Molecular signatures of PV^+^ neurons across brain regions. ***A***, Numbers of differentially expressed genes (*p*_adj_ < 0.01 and |log2FoldDifference| > 1) between cSNAIL-isolated PV^+^ and PV^–^ fractions from the cortex, striatum, and GPe of sham animals (for details, see Extended Data Figure 3-1). ***B***, Normalized read counts from example marker transcripts in each sample. DESeq2 normalized counts were converted to the proportion that they represent of the total read count for that gene within the given brain region. ***C***, Example pathway enrichments for pan-PV^+^ and pan-PV^–^ marker genes (for the full list, see Extended Data Figure 3-4). The size of the bubble represents the negative log of the adjusted *p* value after multiple hypotheses correction. ***D***, Numbers of cell type-enriched ATAC-seq peaks (*p*_adj_ < 0.01 and |log2FoldDifference| > 1) in PV^+^ populations from each brain region (Extended Data Figure 3-2). ***E***, DESeq2 normalized counts of ATAC-seq reads within 5 kb of the TSS of marker genes. Again, counts are represented as the proportion of total counts for that TSS region in the cortex, striatum, or GPe. For the full list of differentially accessible promoters, see Extended Data Figure 3-3. ***F***, Examples of motifs enriched in PV^+^ specific or PV^–^ specific ATAC-seq peaks (Extended Data Figure 3-5). Each brain region was assessed separately, and these motifs were significantly enriched in PV^+^ or PV^–^ sequences across all brain regions.

10.1523/JNEUROSCI.1634-20.2020.f3-1 Figure 3-1 **Differentially expressed genes between** PV^+^ **and** PV^–^ **populations in each brain region.** All genes with DESeq2 *p*_adj_ < 0.01 & |log2FoldDifference| > 1 are reported. Download Figure 3-1, XLSX file.

10.1523/JNEUROSCI.1634-20.2020.f3-2 Figure 3-2 **Differentially accessible ATAC-seq peaks between** PV^+^ **and** PV^–^ **populations in each brain region.** All peaks with DESeq2 *p*_adj_ < 0.01 & |log2FoldDifference| > 1 are reported. Additionally, the corresponding summit-centered 500 bp windows used for motif enrichment analysis are listed for each cell population. Download Figure 3-2, XLSX file.

10.1523/JNEUROSCI.1634-20.2020.f3-3 Figure 3-3 **Genes with differential promoter accessibility between** PV^+^ **and** PV^–^ **populations in each brain region.** Standardized 10 kb windows around the TSS of each gene were assessed for differential chromatin accessibility. Differential TSS regions with *p*_adj_ < 0.1 are reported. Download Figure 3-3, XLSX file.

**Figure 4.**
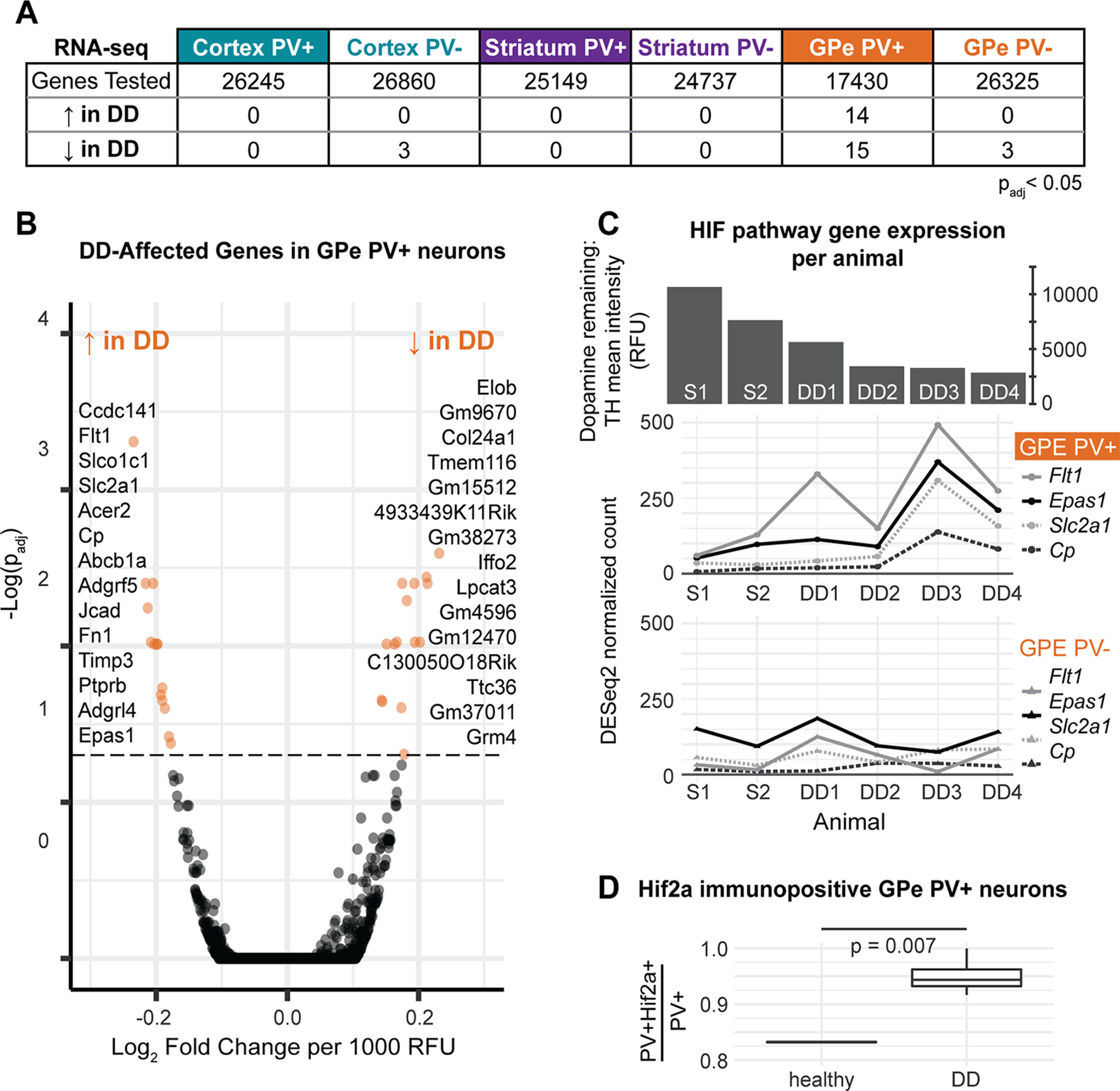
Divergent gene expression in dopamine-depleted mice is largely restricted to GPe PV^+^ neurons and implicates the HIF pathway. ***A***, Numbers of genes that have differential expression with DD in each cell type. DD was confirmed by immunochemistry (Extended Data Figure 4-1), and RNA-seq sample quality was confirmed computationally (Extended Data Figure 4-2). ***B***, Volcano plot of DD-affected genes in GPe PV^+^ neurons. Orange represents genes meeting the significance threshold of *p*_adj_ < 0.05, listed in order of significance. See Extended Data Figure 4-3 for a detailed list of differentially expressed genes and Extended Data Figure 4-5 for functional relationships. ***C***, Dopamine depletion is associated with a specific increase in Hif2a (*Epas1*) transcription and its target genes in GPe PV^+^ neurons. The animals are referred to as S1 and S2 for sham animals and DD1-4 for 6-OHDA-treated animals in order of depletion severity. TH levels are shown for each animal in the bar graph. ***D***, There is an increase in the proportion of GPe PV^+^ cells that express Hif2a protein between images from healthy tissue and DD tissue (*t* test) (Extended Data Figure 4-4).

10.1523/JNEUROSCI.1634-20.2020.f3-4 Figure 3-4 **Functional enrichment of** PV^+^ **and** PV^–^ **enriched genes.** All g:Profiler results with *p*_adj_ < 0.05 for either gene set are shown. Download Figure 3-4, XLSX file.

**Figure 5.**
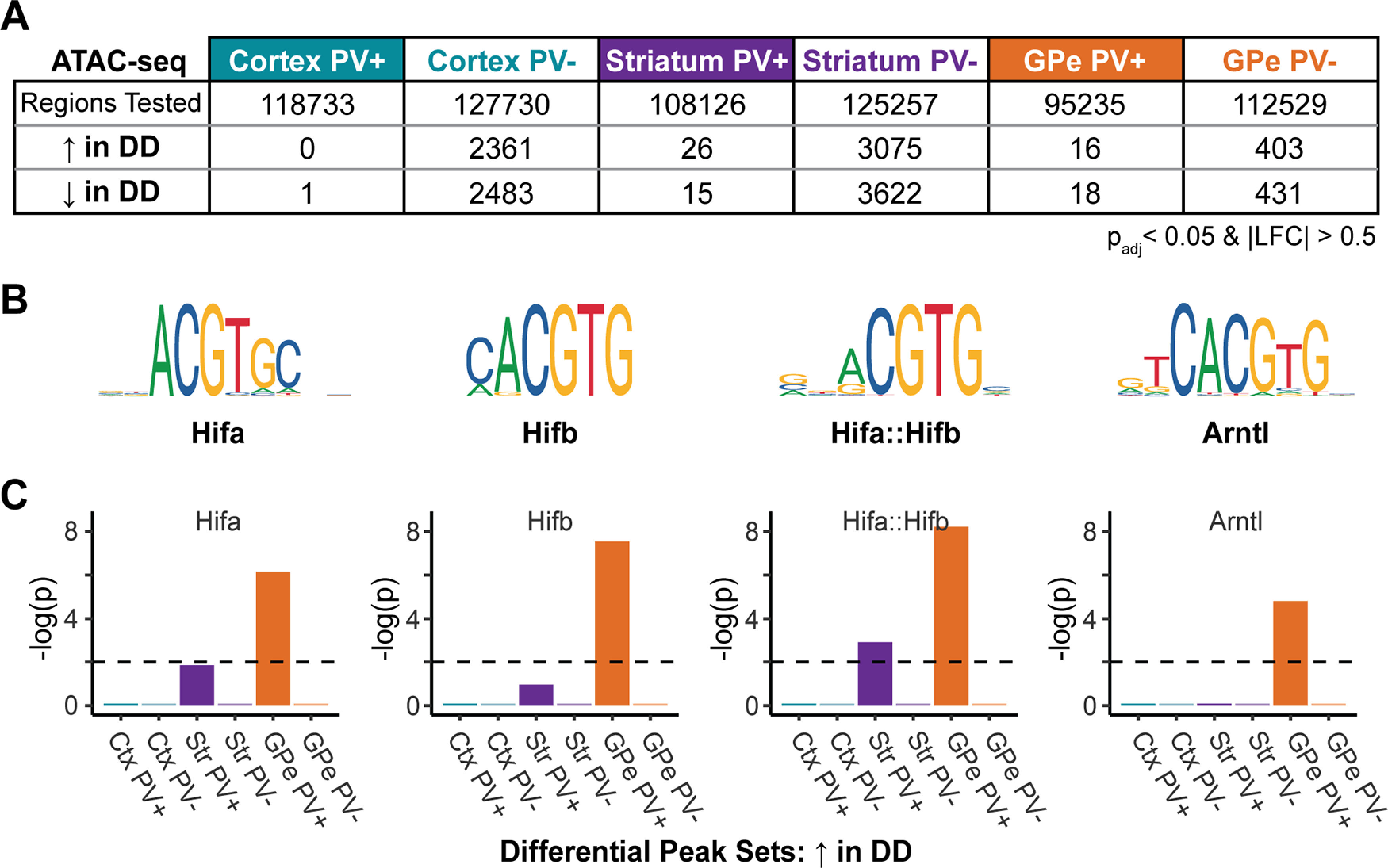
Open chromatin changes associated with DD show cell type-specific HIF motif enrichment. ***A***, Numbers of ATAC-seq regions in each cell type that are differentially open in DD animals compared with sham animals. The data input into these models were high quality (Extended Data Figure 5-1). Full descriptions of differentially accessible regions and potential functions can be found in Extended Data Figures 5-2, 5-3, 5-4, and 5-5. The relationship between TSS accessibility and gene expression in GPe PV^+^ neurons is shown in Extended Data Figure 5-6. ***B***, HIF family transcription factor binding motifs as defined in JASPAR 2018 (Khan et al., 2018). ***C***, Cell type-specific HIF family motif enrichments among sets of sequences underlying ATAC-seq peak summits that increase in accessibility in DD animals. The full set of enriched motifs are available in Extended Data Figure 5-7.

10.1523/JNEUROSCI.1634-20.2020.f3-5 Figure 3-5 **Motif enrichment among** PV^+^ **and** PV^–^ **ATAC-seq peaks.** All AME results with E value < 10 are reported for each analysis. Download Figure 3-5, XLSX file.

10.1523/JNEUROSCI.1634-20.2020.f4-1 Figure 4-1 **Quantification of dopamine depletion per hemisphere. A.** Levels of striatal Tyrosine Hydroxylase for each hemisphere of the four 6-OHDA lesioned animals and 2 sham animals. The p value reflects a significant difference in populations by the standard *t* test. **B.** Examples of the quantified images, representing the two red points in A. Download Figure 4-1, TIF file.

10.1523/JNEUROSCI.1634-20.2020.f4-2 Figure 4-2 **RNA-seq sample similarities. A.** The first two principal components separate samples by brain region and cell type. **B.** In hierarchical clustering, samples mainly cluster by brain region and then cell type. Download Figure 4-2, XLSX file.

10.1523/JNEUROSCI.1634-20.2020.f4-3 Figure 4-3 **Differentially expressed genes with dopamine depletion.** Information for all genes with DESeq2 *p*_adj_ < 0.2 are shown for each cell population. Download Figure 4-3, XLSX file.

10.1523/JNEUROSCI.1634-20.2020.f4-4 Figure 4-4 **Example images of Hif2a staining in healthy and DD GPe tissue**. Some instances of PV^+^Hif2a- cells are highlighted by white arrows. The scale bars measure 100 µm. Download Figure 4-4, TIF file.

10.1523/JNEUROSCI.1634-20.2020.f4-5 Figure 4-5 **Functional enrichment of DD-affected genes in GPe** PV^+^ **neurons**. Enrichments were conducted on the differential gene sets at the relaxed threshold of DESeq2 *p*_adj_ < 0.2 using g:Profiler. All results with *p*_adj_ < 0.05 for either gene set are shown. Download Figure 4-5, XLSX file.

10.1523/JNEUROSCI.1634-20.2020.f5-1 Figure 5-1 **ATAC-seq data are high quality. A & B.** ATAC-seq samples tend to cluster with other samples of the same tissue and cell type by PCA and hierarchical clustering of genome-wide open chromatin profiling. **C.** The data exhibit the characteristic periodicity in fragment length distributions of high quality ATAC-seq data, reflecting nucleosome positioning. The plot shown is a representative example from one GPe PV^+^ sample. **D.** ATAC-seq signal is enriched at transcription start sites (TSS), indicative of high signal-to-noise. The displayed data are a representative example from one GPe PV^+^ sample. Download Figure 5-1, TIF file.

10.1523/JNEUROSCI.1634-20.2020.f5-2 Figure 5-2 **Differential ATAC-seq regions with dopamine depletion.** For PV^+^ cell populations, differential peaks that met the lenient significance of *p*_adj_ < 0.2 are reported. For PV^–^ cell populations, differential peaks that met a significance of *p*_adj_ < 0.05 and |log2FoldChange| > 0.5 are reported. The corresponding summit-centered 500 bp regions for differential motif discovery are also listed. Download Figure 5-2, XLSX file.

10.1523/JNEUROSCI.1634-20.2020.f5-3 Figure 5-3 **Overlap between DD-affected ATAC-seq peaks of different cell types.** The width of each outer segment of the circle shows how many DD-affected peaks were recovered in that cell type. Bidirectional arcs between two cell types signify shared DD-affected peaks where the width of the connection represents the number of shared peaks. Plotted with circlize (Gu et al., 2014). Download Figure 5-3, TIF file.

10.1523/JNEUROSCI.1634-20.2020.f5-4 Figure 5-4 **Summary of enriched annotations in DD-affected ATAC-seq peak sets.** Enrichments were determined using GREAT for each differential set of peaks against a background of all peaks in that cell type. The arrow of a term indicates that it was enriched in DD-increasing peaks (up arrow) or DD-decreasing peaks (down arrow) and the color indicates the cell population. Download Figure 5-4, TIF file.

10.1523/JNEUROSCI.1634-20.2020.f5-5 Figure 5-5 **Enriched annotations in DD-affected ATAC-seq peak sets.** All results from the GREAT analysis with FDR Q < 0.05 and minimum region-based fold enrichment of 2. Download Figure 5-5, XLSX file.

10.1523/JNEUROSCI.1634-20.2020.f5-6 Figure 5-6 **TSS accessibility of DD-affected genes in GPe** PV^+^ **neurons.** The mean of each population is marked with the red star and the reported p values denote the significance of the difference in means by the two-tailed *t* test. Download Figure 5-6, TIF file.

10.1523/JNEUROSCI.1634-20.2020.f5-7 Figure 5-7 **Motif enrichments among DD-affected peak sets.** All AME results with E value < 10 are reported for each analysis. Download Figure 5-7, XLSX file.

##### Vector construction

The modified *Sun1GFP* fusion sequence fragment was synthesized by Integrated DNA Technologies and contained flanking restriction sites for SgsI and BspoI. The *Sun1GFP* sequence was inserted into an AAV DIO backbone vector with an Ef1a promoter using standard restriction cloning. pAAV-Ef1a-DIO-EGFP-WPRE-pA was a gift from Bernardo Sabatini (http://addgene.org/; 37084; RRID:Addgene_37084) (Saunders et al., 2012). The ligation was transformed into electrocompetent bacteria from which we selected positive clones and confirmed the sequence with Sanger sequencing. The cSNAIL genome vector pAAV-Ef1a-DIO-Sun1GFP-WPRE-pA is available on Addgene; RRID:Addgene_160141.

##### AAV production

The AAV was produced by triple cotransfection of pAAV-Ef1a-DIO-Sun1GFP-WPRE-pA, an AAV helper plasmid, and pUCmini-iCAP-PHP.eB into AAVpro(R) 293T cells (Takara, catalog #632273). pUCmini-iCAP-PHP.eB was a gift from Viviana Gradinaru (http://addgene.org/; 103005; RRID:Addgene_103005) (Chan et al., 2017). After cell expansion, the AAV particles were precipitated with polyethylene glycol and purified with ultracentrifugation on an iodixanol gradient. The virus was titrated using the AAVpro(R) Titration Kit (Takara, catalog #6233), diluted in PBS to a concentration of 8.0 × 10^9^ vg/µl, and stored at −80°C until injection.

##### Animals

All molecular experiments were performed on 9- to 12-week-old heterozygous *Pvalb-2A-Cre mice* (B6.Cg-Pvalb^tm1.1(cre)Aibs^/J; Jackson stock #012358) (Madisen et al., 2010) on a C57BL/6J background. Imaging experiments for the validation of cSNAIL (see Fig. 1) were performed with double-transgenic *Pvalb-2A-Cre* and *Ai14* mice (Ai14 strain; B6.Cg-Gt(ROSA)26Sor^tm14(CAG-tdTomato)Hze^/J; Jackson stock #007914) (Madisen et al., 2010). Experiments related to Hif2a staining (see Fig. 4*D*) were conducted on WT mice (C57BL/6J; Jackson stock #000664).

##### AAV delivery

Mice were anesthetized with isoflurane for 2-5 min until breathing slowed and the animal had no pedal reflex. We injected 4 × 10^11^ vg of AAV into the retro-orbital cavity. The mice received 0.5% proparacaine hydrochloride ophthalmic solution for comfort and were monitored for physical and behavioral abnormalities after procedure, but none were observed. The virus incubated for 3–5 weeks to reach peak expression before downstream experiments.

##### Dopamine depletion surgery

The molecular experiments were designed to reproduce previous conditions and maximize cell yield. Therefore, we induced bilateral acute 6-OHDA or saline injections to the medial forebrain bundle as described by Mastro et al. (2017). Tissues were collected for experiments 3–5 d after surgery (Extended Data Fig. 2-1). This time period was chosen to maximize recovery time from the initial injection without allowing the animals to experience long-term discomfort or death because of the resulting neurodegeneration. Throughout the period between surgery and molecular experiments, the animals were closely monitored for weight loss and were provided with warming pads, soft food, and saline injections for hydration.

##### Immunofluorescence staining and imaging

Wherever possible, tissue for imaging was preserved by 4% PFA perfusion. The brains were dissected and incubated overnight at 4°C in 4% PFA. Shortly after, they were vibratome sectioned coronally at 100 µm for antibody staining and slide mounting. To visualize cSNAIL specificity (see Fig. 1*D*), tissues were stained with primary anti-NeuN (Cell Signaling Technology, #12943) and secondary AlexaFluor-405 (Invitrogen, #A-31556) to label neurons. For images related to Figure 4*D* and Extended Data Fig. 4-4, tissues were stained with primary anti-Pvalb (Swant, PV 27) paired with secondary AlexaFluor-405 (Invitrogen, #A-31556) or AlexaFluor-488 (Invitrogen, #A-11034) and primary anti-Hif2a (Novus Biologicals, #NB100-132) paired with secondary AlexaFluor-594 (Cell Signaling Technology, #8890S). For tissues involved in both genomic assays and imaging, one fresh coronal slice, including caudate putamen, but anterior to the GPe, was fixed in 4% PFA for 24 h. Tissues were stained with primary anti-Tyrosine hydroxylase (TH) (Pel-Freez, #P40101-150) and secondary AlexaFluor-647 (Invitrogen, #A-31573) to approximate dopamine levels in the striatum. All imaging sections were mounted onto slides using ProLong Diamond Antifade mounting media (Invitrogen, #P36961) and imaged with confocal microscopy.

##### Image analysis

Single-channel and colabeled cells were manually counted for Figures 1*D* and 4*D*. To quantify the levels of striatal TH as a proxy for remaining dopamine (see Fig. 4*C*), we measured mean pixel intensity with Fiji (Schindelin et al., 2012). Measurements were performed on one 500 × 500 µm square from the striatum of each hemisphere (Extended Data Fig. 4-1), and hemisphere measurements were averaged to determine the TH levels per animal.

##### Nuclei collection

Fresh mouse brain tissue was sliced on a vibratome in cold, oxygenated ACSF. Then, brain ROIs were dissected under a light microscope and transferred into cold ATAC-seq lysis buffer (Buenrostro et al., 2015). The nuclei were extracted from each tissue using 30 strokes of loose pestle Dounce homogenization followed by 70 µm filtration and one centrifugation wash for 10 min at 2000 × *g*.

##### Affinity purification of Sun1GFP^+^ nuclei

The nuclei suspension was precleared for 10-15 min with Protein G Dynabeads (Thermo Fisher Scientific, catalog #10004D) to remove nuclei or debris that had native affinity for the beads. Free nuclei were incubated with anti-GFP antibody (Invitrogen, #G10362) for 30 min before fresh beads were added to the reaction and incubated for another 20 min. All incubations took place at 4°C with 40 rpm end-to-end rotation in wash buffer (0.25 m sucrose, 25 mm KCl, 5 mm MgCl_2_, 20 mm tricine with KOH to pH 7.8, and 0.4% IGEPAL). After this process, the bead-bound nuclei (Sun1GFP^+^ fraction) were purified and washed on a magnet, while the unbound nuclei were spun down and resuspended in water to preserve the Sun1GFP^–^ fraction.

##### RNA-seq library construction

After setting aside 50,000 nuclei for ATAC-seq, we extracted total RNA from all remaining nuclei in each sample using the QIAGEN RNeasy Micro Kit (catalog #74004); 10 pg to 10 ng of total RNA per sample was processed using the Ovation SoLo RNA-seq System with mouse AnyDeplete to remove ribosomal RNA (Tecan, catalog #0501-32). Because many nuclear transcripts are immature, no poly A selection was performed. Libraries were paired-end sequenced at 3-9 million reads per sample on the Nextseq 500 system.

##### RNA-seq processing

The sequencing output files were mapped to the mm10 genome (downloaded from UCSC in November 2015) using Hisat2 with default parameters. Duplicate UMIs were removed using NuDup (https://github.com/tecangenomics/nudup). The quality of the filtered data was assessed using Picard CollectRnaSeqMetrics version 2.8.1 (http://broadinstitute.github.io/picard). A counts per gene per sample matrix was constructed using Subread featureCounts version 1.6.4 (Liao et al., 2019) with GRCm38 gene annotations (Ensembl version 79). Sample similarities were assessed by principal component analysis (PCA) and hierarchical clustering of Euclidean sample distances after log2 transformation with DESeq2's *rlog()* function (Love et al., 2014) (Extended Data Fig. 4-2). The RNA-seq data are available on GEO, accession number GSE157359.

##### Differential gene expression analysis

We conducted two primary gene expression analyses to determine (1) gene expression signatures of Sun1GFP^+^ and Sun1GFP^–^ cells in *Pvalb-2A-Cre* mice under normal dopamine conditions (see Fig. 3; Extended Data Fig. 3-1) and (2) expression changes in six cell populations on DD (see Fig. 4; Extended Data Fig. 4-3). Toward the first aim, differentially expressed genes between Sun1GFP^+^ and Sun1GFP^–^ cells in each brain region were assessed in 2 sham animals. Here, we used the negative binomial model in DESeq2 version 1.14.1 (Love et al., 2014): read count = ∼ sex + Sun1GFP status. For the second analysis, we compared the expression profiles of each cell population between DD and sham mice. Because transcript levels are quantitative, we modeled the RNA-seq counts continuously according to the mean anti-TH fluorescence intensities of each animal, which reflect the extent of DD: DESeq2 read count = ∼ sex + TH intensity. Throughout all analyses, each model was built individually and only included the samples under direct pairwise comparison, ensuring that dispersion estimates were restricted to the relevant samples. We assessed annotation enrichments in differential gene of interest sets using g:Profiler with default parameters (see Fig. 3*C*; Extended Data Figs. 3-4, 4-5) (Raudvere et al., 2019).

##### ATAC-seq library construction

A small subset of nuclei was stained with DAPI, and fluorescent nuclei were manually counted using a hemocytometer to determine their concentration; ∼50,000 nuclei were transferred to a new tube in 22.5 µl water and mixed with 2.5 µl Tagment DNA Enzyme I and 25 µl Tagment DNA Buffer (Illumina, catalog #20034198). The tn5 transposition reaction was conducted at 37°C for 30 min with 300 rpm mixing. After tagmentation, the samples were taken through column purification, amplification, and double-sided size selection (100-1000 bp) with AmpureXP beads (Beckman Coulter, catalog #A63881). To reduce amplification bias, we evaluated a side qPCR reaction for each sample to determine the optimal number of PCR cycles for each sample to reach one-third maximum intensity. To get initial sample quality estimates and balance sample representation for deep sequencing, each sample was sequenced at 1-3 million reads on the Illumina Miseq system. Then, samples were pooled and paired-end sequenced on a Novaseq 6000 to ∼25 million unique, nonmitochondrial reads per sample for analysis.

##### ATAC-seq processing

The fastq files from each sample were processed with the ENCODE ATAC-seq pipeline (https://github.com/kundajelab/atac_dnase_pipelines). Peaks were called on biological replicates of the same cell type, brain region, and treatment with an IDR threshold of 0.1 (https://github.com/nboley/idr). This design maximizes the potential to include peaks that may only be present in one condition while limiting potential false positives that may only be called in one sample. Count tables containing the number of reads per peak for each sample were constructed using Rsubread featureCounts version 1.28.1 (Liao et al., 2019) on the filtered bam alignments. The peak sets for count tables were chosen to reflect the goals of each analysis separately. For analysis of cellular identity (see Figs. 2*B*, 3*D*), the regions tested were the union of IDR peaks from all sham animal samples. For the promoter analysis in Figure 3*E*, instead of ATAC-seq peaks, counts were computed within 10 kb windows around the transcription start sites (TSSs) for all genes assessed in the RNA-seq analysis. To assess DD-affected regions (Fig. 5), six separate count tables were constructed for the six cell populations and represented the union of IDR peaks from sham and DD animals for the given cell type and brain region combination. Finally, to test for a relationship between GPe PV^+^ promoter accessibility and transcription in DD (Extended Data Fig. 5-6), we derived a count table for all GPe PV^+^ ATAC-seq samples within 2 kb windows around each TSS. Sample similarities were assessed among all ATAC-seq samples by PCA and hierarchical clustering using DESeq2 on a count table across the merged union of peaks from all samples (Extended Data Fig. 5-1*A*,*B*). Additionally, we ensured that each sample had TSS signal enrichment and exhibited the characteristic fragment length periodicity of high-quality ATAC-seq data, reflective of nucleosome positioning (Extended Data Fig. 5-1*C*,*D*). The ATAC-seq data are available on GEO, accession number GSE157359.

##### Differential open chromatin analysis

In parallel to the RNA-seq analysis, we assessed differentially accessible chromatin regions between Sun1GFP^+^ and Sun1GFP^–^ fractions in sham animal samples and regions that were affected by DD in each cell type. Sun1GFP^+^ and Sun1GFP^–^ comparisons in each brain region were assessed in DESeq2 version 1.14.1 (Love et al., 2014) with read count = ∼ sex + Sun1GFP status (see Figs. 2*B*, 3*D*,*E*; Extended Data Figs. 3-2, 3-3). For DD-affected open chromatin analyses (see Fig. 5; Extended Data Figs. 5-2, 5-6), we used a binary model of treatment state, DD versus saline, controlling for sex differences: read count = ∼ sex + treatment. Again, models for each cell population were built separately and only included samples for one comparison. Differential ATAC-seq peak sets of interest were subjected to pathway enrichment analysis using GREAT version 3.0.0 (Extended Data Figs. 5-4, 5-5) (McLean et al., 2010).

##### Motif enrichment analysis

Differential motif enrichment analyses (see Figs. 3*F*, 5*C*; Extended Data Figs. 3-5, 5-7) were performed using AME version 5.0.5 (Mcleay and Bailey, 2010) with motifs from the nonredundant JASPAR 2018 core vertebrates database (Khan et al., 2018). First, peak sets of interest were summit-centered and unified to 500 bp. Where more than one summit was called within a peak and these summits were >100 bp apart, we included both entries in the analysis. For Figure 3*F*, enrichments were assessed relative to the peaks enriched in the other cell type of that tissue (e.g., cortex PV^+^ enriched peaks were tested for motif enrichments relative to cortex PV^–^ enriched peaks). For Figure 5*C*, enrichments were assessed relative to a background of all IDR peaks called in cells of the same brain region and Sun1GFP fraction as the foreground.

##### Single nucleus (sn)ATAC-seq processing

To compare cSNAIL-isolated cell types with single nucleus sequencing cluster markers, we reprocessed a publicly available snATAC-seq dataset from adult mouse cortex, provided by 10× Genomics. The data were downloaded and processed following the Signac adult mouse brain vignette (https://github.com/timoast/signac). We defined peaks that marked each cluster as those with log2FoldDifference > 0.25 and *p* < 0.05 relative to all other clusters.

##### snRNA-seq processing

Raw count matrices derived from 10× chromium v3 experiments conducted in mouse primary motor cortex were downloaded from NEMO (https://portal.nemoarchive.org, samples PBICCNSMMRMOPI70470511BD180328, PBICCNSMMRMOPI7047059TD180328, PBICCNSMMRMOPI70470512BD180328B, PBICCNSMMRMOPI70470512BD180328C, PBICCNSMMRMOPI70470510TD180328, and PBICCNSMMRMOPI70470512BD180328A). Empty droplets were removed using the DropletUtils package (Griffiths et al., 2018) *defaultDrops* method. Doublets were predicted and removed using the SCDS package (Bais and Kostka, 2019) *cxds_bcds_hybrid* method with a score cutoff of 1.0. The UMI count values were normalized using pooled size factor normalization (Lun et al., 2016a) implemented in the Scran package (Lun et al., 2016b). Size factor normalization requires a preclustering step, which was computed using the Scanpy (Wolf et al., 2018) package's Leiden community detection algorithm (Traag et al., 2019). To create the UMAP (McInnes et al., 2018) for visualization, we identified the top 7500 most variable genes using *scanpy.pp.highly_variable_genes* with flavor “seurat” and computed the principle components on these genes with *scanpy.pp.pca*. The UMAP was then computed based on the PCA reduced single nucleus gene expression matrix. To compute a PV gene signature score for each cell, we averaged the PV^+^ positively differentially expressed genes in the scran normalized, mean centered, and SD scaled gene expression matrices and subtracted the averaged PV^–^ positively differentially expressed genes in the same matrices.

##### Statistical analyses

To determine the specificity and efficiency of cSNAIL labeling in each brain region (Fig. 1*E*), these metrics were quantified for four independent images and averaged. This approach is more appropriate than computing the specificity on the pool of all cells together because treating images as replicates allows us to also quantify variation in the data. To this end, the SEM across the four images is reported for each brain region. The same principles were used for the quantification of TH (Extended Data Fig. 4-1) and Hif2a^+^ PV^+^ neurons on DD (Fig. 4*D*). Images from tissue of the same treatment state (DD or no DD) were treated as replicates and the spread of the data are shown by the box plot. To test for a difference between the means of the two treatment groups, we used two-tailed *t* test statistics.

For comparing cSNAIL ATAC-seq markers to snATAC-seq cluster markers (Fig. 2*B*), we used the one-sided hypergeometric enrichment test, sampling cell type-specific cSNAIL peaks in each brain region from an expected population frequency of cluster-specific snATAC-seq peaks. The hypergeometric distribution is commonly used to test for association of binary variables in genomics and is suitable to help us interrogate which clusters have the most in common with cSNAIL-isolated cells. Only peaks present in both the cSNAIL-isolated dataset and the snATAC-seq dataset (*N* = 112,082) were evaluated. For simplicity, cluster-specific peaks from all excitatory neuron clusters were combined into one category. For visualization, we converted the hypergeometric test enrichments throughout each cSNAIL cell population into *z* scores and plotted them using gplots heatmap.2 in R (https://github.com/talgalili/gplots).

In order to visualize the extent to which each snRNA-seq nucleus had a similar expression profile to cSNAIL-isolated nuclei (Fig. 2*C*), we computed a per-nucleus gene signature score that is the difference between the normalized expression of Sun1GFP^+^ specific genes and Sun1GFP^–^ specific genes. Because the snRNA-seq came from the healthy cortical tissue, we focused on genes that were differentially expressed between sham cortical Sun1GFP^+^ and Sun1GFP^–^ fractions. The Sun1GFP^+^ and Sun1GFP^–^ scores were computed by averaging across the scaled (0 mean centered, unit variance) expression of signature genes across all cells.

For differential gene expression and differential chromatin accessibility analyses (see Figs. 3–5), genes or ATAC-seq regions were assessed using the negative binomial model in DESeq2 version 1.14.1 with Wald test statistics. The parameters and inputs of models were tailored to each analysis (see sections above for specific model formulas), but all models included data from both sexes and controlled for sex differences. The significance of differential genes or regions was evaluated using the log2 fold difference between two cell types or conditions and the *p* value after multiple hypothesis correction with the Benjamini Hochberg method (*p*_adj_). Significance cutoffs for specific analyses are described throughout Results, and the statistics for these tests can be found in Extended Data Figs. 3-1, 3-2, 3-3, 4-3, and 5-2.

To assess concordance between gene transcriptomic and epigenomic changes in GPe PV^+^ neurons on DD (Extended Data Fig. 5-6), we categorized genes into nonaffected, DD-increasing, or DD-decreasing and plotted the log2 fold difference of ATAC-seq signal within a 2 kb window around the TSS of these genes. We tested for a difference in population means between these groups using the two-tailed *t* test statistics.

Gene annotation enrichment analyses are a similar statistical problem as the snATAC-seq cluster marker .comparison in that the objective is to test for overrepresentation of features belonging to a certain category within a feature set of interest. Therefore, we also used the hypergeometric test in this instance, as implemented by g:Profiler (Raudvere et al., 2019) (see Fig. 3*C*; Extended Data Figs. 3-4, 4-5). Similarly, we used hypergeometric test statistics as implemented in GREAT version 3.0.0 (McLean et al., 2010) to explore enriched pathways associated with differential ATAC-seq peak sets of interest (Extended Data Figs. 5-4, 5-5).

Motif enrichment analyses in AME (Mcleay and Bailey, 2010) (Figs. 3*F*, 5*C*; Extended Data Figs. 3-5, 5-7) were conducted using the average odds scoring method, which calculates the average position weight matrix motif score for each input sequence, and Fisher's exact test statistics. The reported *p* values were adjusted for multiple hypothesis testing using a Bonferroni correction.

## Results

### cSNAIL isolates Cre^+^ nuclei with high precision

cSNAIL technology is a viral strategy for labeling and isolating the nuclei of Cre-expressing cells. To achieve nuclei isolation compatible with epigenomic profiling, we used a similar strategy to the Sun1GFP INTACT transgenic mouse strain (Mo et al., 2015). Sun1 is a highly conserved nuclear envelope protein that helps connect the nucleoskeleton and the cytoskeleton (Haque et al., 2010). Because of its tight association with the inner nuclear envelope, the Sun1GFP fusion protein localizes the GFP protein to the nuclear surface. This positioning allows for the affinity purification of nuclei, whereby after tissue is homogenized into a single-nuclei suspension, magnetic beads coated with anti-GFP antibody specifically bind Sun1GFP^+^ nuclei, and they can be separated from Sun1GFP^–^ nuclei with a magnet. Faced with the small capacity of AAV, we made several modifications to the INTACT mouse transgene design (see Materials and Methods). The resulting cSNAIL genome expresses a modified Sun1GFP protein in a Cre-dependent manner. We packaged the cSNAIL Sun1GFP vector into AAV variant PHP.eB, which is capable of crossing the blood-brain barrier to broadly transduce the CNS (Chan et al., 2017).

We evaluated the ability of cSNAIL to drive Cre-specific nuclear anchored independent labeling in the *Pvalb-2A-Cre* mouse line with imaging and molecular data (Figs. 1, 2). With fluorescent imaging, we confirmed that cSNAIL promotes Sun1GFP expression that is properly localized to the nuclear envelope in tissue (Fig. 1*B*). This expression is sufficient to bind anti-GFP coated magnetic beads after tissue dissociation (Fig. 1*C*). To quantify the ability of cSNAIL to correctly target Cre^+^ cells, we compared the expression of cSNAIL to the expression of Cre reporter tdTomato of the *Pvalb-2A-Cre/Ai14* mouse strain (Fig. 1*D*,*E*). For the purposes of this analysis, we defined virus specificity as the percent of Sun1GFP^+^ cells that also express tdTomato and virus efficiency as the percent of tdTomato^+^ cells that also express Sun1GFP. cSNAIL exhibited very strong (>94%) specificity in the cortex, striatum, and GPe. The efficiency ranged from 53% to 81%, which reflects the expected transduction of the viral capsid in these brain regions (Chan et al., 2017).

To confirm the molecular identity of cSNAIL-isolated nuclei from *Pvalb-2A-Cre* mice, we compared ATAC-seq and RNA-seq from sham animal Sun1GFP^+^ and Sun1GFP^–^ fractions to publicly available PV^+^ and PV^–^ data. cSNAIL ATAC-seq and RNA-seq exhibited the expected cell type-specific patterns at the *Pvalb* locus compared with signals from INTACT-isolated populations (Mo et al., 2015) (Fig. 2*A*). When comparing our cell type-specific ATAC-seq markers to single nucleus (sn)ATAC-seq clusters from the adult mouse cortex (https://github.com/timoast/signac), the Sun1GFP^+^ peaks from each brain region were enriched for PV^+^ cluster markers (Fig. 2*B*). Aligning with the expected cell type proportions in each brain region, Sun1GFP^–^ fractions were most enriched for snATAC-seq markers of excitatory neuron clusters (cortex only) and glial clusters.

We focused on marker open chromatin peaks from clusters of cells because of the sparse, generally binary nature of snATAC-seq counts. On the other hand, the quantitative properties of snRNA-seq transcript counts enable a richer interpretation of cSNAIL signatures at the level of individual nuclei. Taking advantage of this, we explored which snRNA-seq nuclei from the mouse primary motor cortex had similar expression patterns to our cortical cSNAIL-isolated populations. First, we defined cSNAIL Sun1GFP^+^ and cSNAIL Sun1GFP^–^ gene expression signatures in the cortex (i.e., strongly differentially expressed genes) (Extended Data Fig. 3-1). Then we scored snRNA-seq nuclei based on the difference between their expression of the cSNAIL Sun1GFP^+^ and cSNAIL Sun1GFP^–^ gene signatures (Fig. 2*C*; Extended Data Fig. 2-2). As expected, nuclei in the PV^+^ neuron cluster had the strongest expression of the cSNAIL Sun1GFP^+^ gene signature, followed by other inhibitory neuron clusters. All excitatory neuron and non-neuronal clusters tended to contain cells with dominating cSNAIL Sun1GFP^–^ signatures. Together, these comparisons provide evidence that cSNAIL Sun1GFP^+^ samples are indeed strongly enriched for PV^+^ neurons. Therefore, throughout the remainder of this paper, we refer to cSNAIL Sun1GFP^+^ and Sun1GFP^–^ cells as PV^+^ and PV^–^ cells, respectively.

### Molecular signatures of cSNAIL-isolated PV^+^ and PV^–^ cells

In each brain region, we recovered hundreds of differentially expressed genes and thousands of differentially accessible ATAC-seq regions between PV^+^ and PV^–^ cell populations (Fig. 3*A*,*D*; Extended Data Figs. 3-1, 3-2). We defined 35 PV^+^ and 29 PV^–^ marker genes with high specificity (DESeq2 *p*_adj_ < 0.01 and |log2FoldChange| > 1) across all three tissues. Of these genes, a small number also had corresponding cell type-specific chromatin accessibility within a 10 kb window around the gene TSS (Extended Data Fig. 3-3), including those highlighted in Figure 3*B*, *E*. Functional gene enrichment analysis on pan-PV^+^ and PV^–^ differential genes implicated neuron-related pathways among the PV^+^ signature and glial pathways, especially oligodendrocyte-related pathways, among the PV^–^ signature (Fig. 3*C*; Extended Data Fig. 3-4). This was not surprising given that neuron composition captured in PV^–^ fractions varies greatly between the cortex, striatum, and GPe.

Next, we sought to identify transcription factor binding motifs that could play a role in epigenetically defining PV^+^ neurons. We tested for enriched motifs in sequences underlying PV^+^ and PV^–^ specific ATAC-seq peaks in each brain region (Fig. 3*F*; Extended Data Fig. 3-5). Among the significantly enriched PV^+^ motifs in all three brain regions was Tcef3, which has been previously implicated in PV^+^ neuron-specific gene regulation (Mo et al., 2015). Additionally, we determined consistent PV^+^ enrichment for the Esrrg and Hoxa2 motifs. The Esrrg transcript itself was also upregulated in PV^+^ neurons in all three brain regions (*p*_adj_ < 0.001), but Tcef3 or Hoxa2 were not. Consistent PV^–^ motif enrichments across all brain regions included the motif for Sox10, which is necessary for the differentiation and survival of oligodendrocytes (Pozniak et al., 2010; Takada et al., 2010). There were also significant enrichments for many other Sox family motifs and the Meis1 motif among PV^–^ ATAC-seq sequences (Extended Data Fig. 3-5). The representation of glial signatures among PV^–^ cell ATAC-seq and RNA-seq indicates that our nuclei dissociation during cSNAIL affinity purification is not restricted to neurons.

### Gene expression differences in dopamine-depleted animals

Severe DD was induced with bilateral acute 6-OHDA lesions, while control animals received a sham surgery of a saline injection under the same conditions. Molecular experiments were conducted 3-5 d after surgery to precisely replicate the timing when GPe PV^+^ neuron stimulation is behaviorally significant (Mastro et al., 2017). All DD experiments were conducted when the mice were heavily symptomatic, before reaching the point of death. This paradigm specifically captures the advanced rodent DD phenotype, which is physiologically similar whether the DD was acute or gradual (Willard et al., 2019). Striatal tissue from 6-OHDA-lesioned animals had significantly lower levels of dopamine production enzyme TH than sham animals, indicating successful depletions (Extended Data Fig. 4-1). All depletions were bilateral, except in 1 animal, DD2, where one hemisphere was only weakly depleted. The RNA-seq data were high quality for all samples except one striatum PV^–^ sample from a DD animal, which was excluded from the analysis. Samples separated by tissue and cell type in PCA and hierarchical clustering, indicating higher variability between cell types than between biological replicates (Extended Data Fig. 4-2).

In the RNA-seq data from DD and sham animals, we recovered very few significant DD-affected genes in any cortical cell populations, striatal cell populations, or in the GPe PV^–^ population. In contrast, GPe PV^+^ neurons contained 29 differentially expressed genes in DD (*p*_adj_ < 0.05) (Fig. 4*A*,*B*; Extended Data Fig. 4-3). It was readily apparent that there were many genes involved in cellular oxygen homeostasis and neuroprotection among the 14 genes that were upregulated in the GPe PV^+^ neurons of DD animals. These included *Epas1*, which encodes for Hypoxia-inducible factor 2α (transcription factor Hif2a), and three of its targets: *Cp*, *Slc2a1*, and *Flt1* (Schofield and Ratcliffe, 2004; Dengler et al., 2014; Smeyne et al., 2015). Additionally, the downregulated set included *Elob*, a moderator of free oxygen sensing that inhibits Hif2a activity (Ohh et al., 2000). These results suggest that GPe PV^+^ neurons may react to DD through hypoxia-inducible factor (HIF) signaling and associated transcriptional responses.

To see whether increased *Epas1* transcription in GPe PV^+^ neurons was accompanied by increased Hif2a protein levels, we performed double immunofluorescent staining for Pvalb and Hif2a in healthy and DD mouse tissue. Indeed, a higher proportion of GPe PV^+^ neurons expressed Hif2a in images from DD animals compared with healthy animals (Fig. 4*D*; Extended Data Fig. 4-4). The change in mean proportion was 11.3%, and this difference was significant (*t* test, *p* = 0.007).

Other differentially expressed genes in the GPe PV^+^ neurons of DD mice have also been implicated in processes of neurodegeneration and neuroprotection. For example, we observed overexpression of *Timp3*, a key inhibitor of matrix metalloproteinases which contribute to dopaminergic neuron apoptosis in PD (Kim et al., 2007). *Timp3* expression in neurons is protective against blood-brain barrier damage, but high levels of *Timp3* can lead to cell death (Rosenberg, 2009). *Lpcat3*, a gene we found to have reduced expression in GPe PV^+^ neurons on DD, is necessary for ferroptosis, which has been linked to neurodegeneration in PD and Alzheimer's disease (Stockwell et al., 2017). Because of the small number of regulated genes, functional enrichment for specific pathways was low (Extended Data Fig. 4-5).

### Open chromatin differences in dopamine-depleted animals

In addition to transcriptome profiling, the biology of epigenetics suggests that it could provide valuable information about the transcriptional network activated during DD. In contrast to gene expression, the epigenetic state of a cell can be more reflective of its past history as well as its future potential to respond to a stimulus (West et al., 2001; Day and Sweatt, 2011). For example, epithelial stem cells retain a “memory” of acute stress in their chromatin, which later influences the genes that are expressed in response to a secondary challenge (Naik et al., 2017). This is also true in the brain, where the epigenetic state of a brain region, and even specific cell types, is highly correlated with how a gene responds to neural activity (Spiegel et al., 2014; Whitney et al., 2014). DNA methylation and histone modification differences have been described in PD, mostly in blood and the SNpc, suggesting that epigenetic gene regulation may be important in this context (van Heesbeen and Smidt, 2019). In this study, we compared the chromatin landscapes between DD and sham animals in each cell population with ATAC-seq, a measurement that is correlated with *cis* regulatory element activity (Buenrostro et al., 2013).

Based on standard QC metrics (see Materials and Methods), the ATAC-seq data were high quality for all samples, except one cortex PV^+^ sample from a DD animal, which was excluded from the analysis (Extended Data Fig. 5-1). In DD animals, we observed a small number of confident open chromatin changes in PV^+^ cell types and many changes within PV^–^ cell types that met significance at a cutoff of *p*_adj_ < 0.05 (Fig. 5*A*; Extended Data Fig. 5-2). The DD-affected open chromatin regions in different cell types had some redundancy, but the majority were cell type-specific at the level of the individual regulatory element (Extended Data Fig. 5-3) (Gu et al., 2014). However, DD-affected regions in multiple cell types tended to converge around areas of the genome enriched for certain functions, including neurotransmission, immune response, and the methionine cycle (Extended Data Figs. 5-4, 5-5).

We then assessed whether the observed differences in open chromatin could be responsible for the transcriptional differences observed in the GPe PV^+^ population (Extended Data Fig. 5-6). Genes with increased expression in DD on average had higher levels of open chromatin at their promoters, but the shift in means was not significant. As a population, genes that decreased in expression on DD had no DD-associated shift in promoter accessibility relative to nonaffected genes. Additionally, there were no overlaps in the most significantly differential genes and peaks. These findings suggest that the mechanisms of gene regulation in this case may favor distal *cis* interactions and *trans* factors.

Because of the increased expression of HIF-related transcripts in GPe PV^+^ neurons in DD, we hypothesized that changing open chromatin regions in this cell type would contain HIF transcription factor binding motif sites. Upon activation, Hifa subunits heterodimerize with Hifb and the complex binds regulatory elements in the genome to affect transcription. Therefore, we conducted a motif enrichment analysis for four HIF family motifs with Fisher's exact test statistics. These included the JASPAR 2018 motifs (Khan et al., 2018) for (1) Hif1a (MA1106.1), which has core motif redundancy with Hif2a (Schödel et al., 2011), (2) Arnt or Hif1b (MA0004.1), (3) the Hifa-Hifb complex (MA0259.1), and (4) a related Hifa binding partner Arntl (M0603.1) (Fig. 5*B*). Because there were too few examples of DD-affected PV^+^ peaks for statistical enrichment analysis at this threshold, we assessed PV^+^ enrichments within peaks that met a more lenient threshold of *p*_adj_ < 0.2 (Extended Data Fig. 5-2). As predicted, all four motifs were highly enriched within GPe PV^+^ DD-increasing peaks (i.e., the set of ATAC-seq peaks with increased accessibility in on DD) (Fig. 5*C*). Moreover, the motifs were not enriched in DD-increasing peak sets from other cell types with the exception of some weaker enrichment in striatal PV^+^ neurons. Finally, sets of ATAC-seq peaks with decreased accessibility in DD (DD-decreasing) were not enriched for HIF motifs in any cell type, including GPe PV^+^. These results indicate a cell type-specific induction of HIF signaling in GPe PV^+^ neurons on DD.

To assess which additional transcription factors may be involved in the epigenetic response to DD, we extended the analysis to all motifs within the nonredundant JASPAR 2018 core vertebrates database (Khan et al., 2018) (Extended Data Fig. 5-7). Specific to DD-increasing GPe PV^+^ peaks and, to a lesser extent, also DD-increasing striatal PV^+^ peaks, we observed an enrichment for Ets-related transcription factor motifs, including Gabpa (GPe *p*_adj_ = 1.25e-30, striatum *p*_adj_ = 1.41e-10). Gabpa is an essential regulator of many cellular respiration genes, including multiple cytochrome *c* oxidase subunits (Ongwijitwat et al., 2006).

DD-affected open chromatin regions in other cell types contained enrichments for several additional transcription factor binding motifs. Notably, DD-increasing peak sets in every cell population, except for GPe PV^+^, were all enriched for the binding motifs of glucocorticoid receptor transcription factors Nr3c1, Nr3c2, and Ar (*p*_adj_ < 0.01). Glucocorticoid receptors are emerging as key regulators of neuroinflammation, and disruption of their proper regulation is thought to play a role in the onset of PD by allowing the infiltration of cytotoxins in the SNpc (Herrero et al., 2015). Our findings are consistent with similar stressors in the cortex, striatum, and GPe.

## Discussion

We began with a query on the cell type-specific molecular changes in GPe PV^+^ neurons of 6-OHDA-treated DD mice. cSNAIL technology enabled the efficient isolation of this precise cell population and the others in these experiments. Because cSNAIL is delivered through AAV, we were able to conduct the molecular experiments on single transgenic animals instead of double transgenics, reducing animal use. Furthermore, cSNAIL packaging in the PHP.eB capsid enabled whole-brain transduction on intravenous injection, maximizing cell yield from each individual and minimizing the confounding inflammation associated with direct injection to the brain. The cSNAIL labeling and isolation system is immediately compatible with most available Cre transgenic strains.

Compared with other technologies, nuclear surface labeling strategies, such as INTACT, are uniquely well suited for the isolation of rare cell types for genomic and epigenetic assays. cSNAIL marks an important advance in cell isolation because it evolves the advantages of immunopurification tagging into a system that is much more flexible and scalable. The Sun1GFP peptide design in cSNAIL is the first demonstration of a nuclear surface tag gene that is compact enough to be packaged in AAV. This Sun1GFP variant can be easily incorporated into other constructs to achieve unprecedented access to small cell populations in a strain-independent manner. For example, the *Sun1GFP* gene could be expressed under the control of a cell type, region, or condition-specific regulatory element (Blankvoort et al., 2018). Unlike the generation of new transgenic lines, new cSNAIL adaptations can be simply created through vector cloning and have the potential to be transferrable across species. In the future, cSNAIL may also be paired with snRNA-seq and snATAC-seq to conduct more detailed profiling of molecular diversity within a genetically defined cell population.

Using cSNAIL technology in *Pvalb-2A-Cre* mice, we characterized the defining transcriptional and epigenomic features of PV^+^ neurons across the cortex, striatum, and GPe. It is important to note that this implementation of cell sorting with cSNAIL was Cre-dependent. Thus, these populations directly reflect the cells captured in this Cre mouse strain, which may differ from other definitions of PV^+^ neurons. Additionally, because we compared with PV^–^ cell population data and not other isolated subtypes, some PV^+^ enriched signatures may be general features of inhibitory neurons and not necessarily restricted to PV^+^ neurons.

Consistent with previous electrophysiological observations and interventions, we found that GPe PV^+^ neurons were a critically affected population in the DD brain. Indeed, in this paradigm, GPe PV^+^ neurons were the only cell population with notable gene expression changes after DD out of the six included cell populations. This is in contrast to some previous reports describing transcriptional alterations in the striatum in PD brain tissue (Miller et al., 2006; Botta-or et al., 2012). While acute animal models are not sufficient to capture the complexities of human PD, they are valuable for understanding some specific consequences of dopamine depletion in a direct manner. Certain other animal models of DD, including MPTP lesion and levodopa-induced models, also produce striatal transcription changes (Chin et al., 2008; Heiman et al., 2014). In this case, we selected the specific paradigm we used to best answer our hypothesis about GPe PV^+^ neurons in late-stage DD. We acknowledge that other animal models may be better suited for striatum-specific inquiries or to examine timing-specific effects throughout the progression of PD. In this severe, acute model of DD, we found evidence that the HIF signaling pathway, part of the cellular oxidative stress response, is induced in GPe PV^+^ neurons. These results implicate new molecular changes within the neural circuit for movement production, but outside the SNpc, as a consequence of DD.

Oxidative stress is known to be an important factor for the degeneration of dopaminergic neurons during the onset of PD and is thought to be a consequence of mitochondrial dysfunction. Postmortem SNpc tissue from idiopathic PD patients contains deficits in mitochondrial complex I of the electron transport chain (Schapira et al., 1990). This is also the specific deficit caused by the MPTP animal model of PD (Langston et al., 1984; Heikkila et al., 1989; Schapira et al., 1990). Other cellular and animal models of PD indirectly compromise mitochondrial activities, including 6-OHDA induction (Blum et al., 2001; Hunter et al., 2007; Xie and Chung, 2012). Moreover, major PD risk alleles affect mitochondrial genes and genes involved in mitochondrial function, including PINK1, PRKN, DJ-1, and SNCA, among others (Larsen et al., 2018).

Previous transcriptomic assays have reported RNA abnormalities in PD related to mitochondrial dysfunction and oxidative stress in the SNpc and striatum of mice treated with MPTP, methamphetamine, or mutated α synuclein (Chin et al., 2008; Soreq et al., 2012). Although we did not recover transcriptomic evidence of oxidative stress in the striatum, our results support the emerging hypothesis that cellular oxygen availability influences not only the onset of PD in the SNpc, but also the downstream circuit. It is unclear whether the HIF response we observed in GPe PV^+^ neurons is a sign of cellular distress, a neuroprotective device, or both. In the SNpc, Hif2a is necessary for adult dopaminergic neuron function and is neuroprotective against dopaminergic neuron loss (Smeyne et al., 2015). There have been reports of GPe PV^+^ specific neurodegeneration in some more gradual models of DD (Hardman and Halliday, 1999; Fernández-Suárez et al., 2012), but little is known about their longevity with respect to other neurons in acute DD implementations. There may be opportunities for gene therapies involving the moderation of oxygen use in these cells, but further work is necessary to discern the potential here.

Why would transcriptional responses to DD manifest selectively in GPe PV^+^ neurons over other populations in the basal ganglia? Fast-spiking PV^+^ neurons have high-energy demands compared with other types of neurons and are therefore more susceptible to fluctuations in oxygen availability or mitochondrial function (Kann, 2016). Disruption in cellular respiration in PV^+^ neurons, especially during development, is an immense burden *in vivo* and has been linked to neuropsychiatric disorders, including schizophrenia and autism spectrum disorder (Inan et al., 2016; Steullet et al., 2017). Impaired mitochondrial function in PV^+^ neurons is associated with changes in synaptic input onto PV^+^ neurons, PV^+^ neuron excitability, and network oscillations (Inan et al., 2016). Because neuronal activity itself can have drastic impacts on gene regulation (West et al., 2001), the observed transcriptional changes in GPe PV^+^ neurons in DD could also be a consequence of changes in circuit properties.

Unlike the specific nature of the transcriptional response to DD, the open chromatin data suggest more global epigenetic effects. These open chromatin observations could be a signature of an epigenetic “scar,” where DD temporarily disrupted gene expression and an epigenomic response restored gene expression to near normal levels, but the open chromatin state remains altered (Maze et al., 2014). Under some circumstances, Hif2a has been shown to autoregulate, whereby increased Hif2a protein levels promote additional transcription of the *Epas1* gene (Sato et al., 2002). This provides a possible mechanism for the amplification and persistence of HIF network genes while other acute transcriptional responses to DD subside. Another consideration for the apparent dissonance between substantial epigenome alterations with no transcriptome alterations in PV^–^ cells is the locations of the differential ATAC-seq peaks. In PV^–^ cell types, the vast majority of differential ATAC-seq peaks occur at distal regulatory elements further than 2000 bp away from a TSS (cortex = 95.9%, striatum = 94.1%, GPe = 95.3%). Changes in accessibility at distal regulatory elements may or may not confer an appreciable change in gene regulation.

Nonetheless, the changes in open chromatin within GPe PV^+^ neurons corroborate and strengthen the HIF upregulation that we observed at the transcript level. Namely, HIF family motifs are selectively enriched within DD-increasing ATAC-seq peaks in GPe PV^+^ neurons. Finally, a higher proportion of GPe PV^+^ neurons appear to have high Hif2a protein levels in DD tissue compared with healthy tissue. Together, the evidence strongly supports an increase in HIF transcription factor activity in GPe PV^+^ neurons on DD. In the future, cSNAIL could be paired with protein assays to determine the precise HIF protein expression levels, cellular localizations, and DNA binding events in GPe PV^+^ neurons in DD.

In conclusion, cSNAIL is a reliable new strategy for labeling and isolating nuclei of a specific subset of cells within cell type-specific Cre animals. In the case of GPe PV^+^ neurons in DD mice, cSNAIL enabled targeted transcriptomic and epigenetic interrogation and yielded new cell type-specific results. Most notably, we recovered evidence of HIF oxidative stress signaling that was specific to GPe PV^+^ neurons in this model of DD. These results may help explain the pathogenic progression of DD throughout the basal ganglia and provide hypotheses toward new cell type-specific interventions in PD. We believe a neuron subtype aware approach, such as cSNAIL, will be necessary to tease out molecular correlates of many neurologic and psychiatric disorders, especially where rare neuron populations are heavily implicated.
