# Supplementary figures and images for: Cell Type-Specific Oxidative Stress Genomic Signatures in the Globus Pallidus of Dopamine-Depleted Mice
Source: J Neurosci. 2020 Dec 9;40(50):9772–83. doi: 10.1523/JNEUROSCI.1634-20.2020 (PMC7726543; doi:10.1523/JNEUROSCI.1634-20.2020)

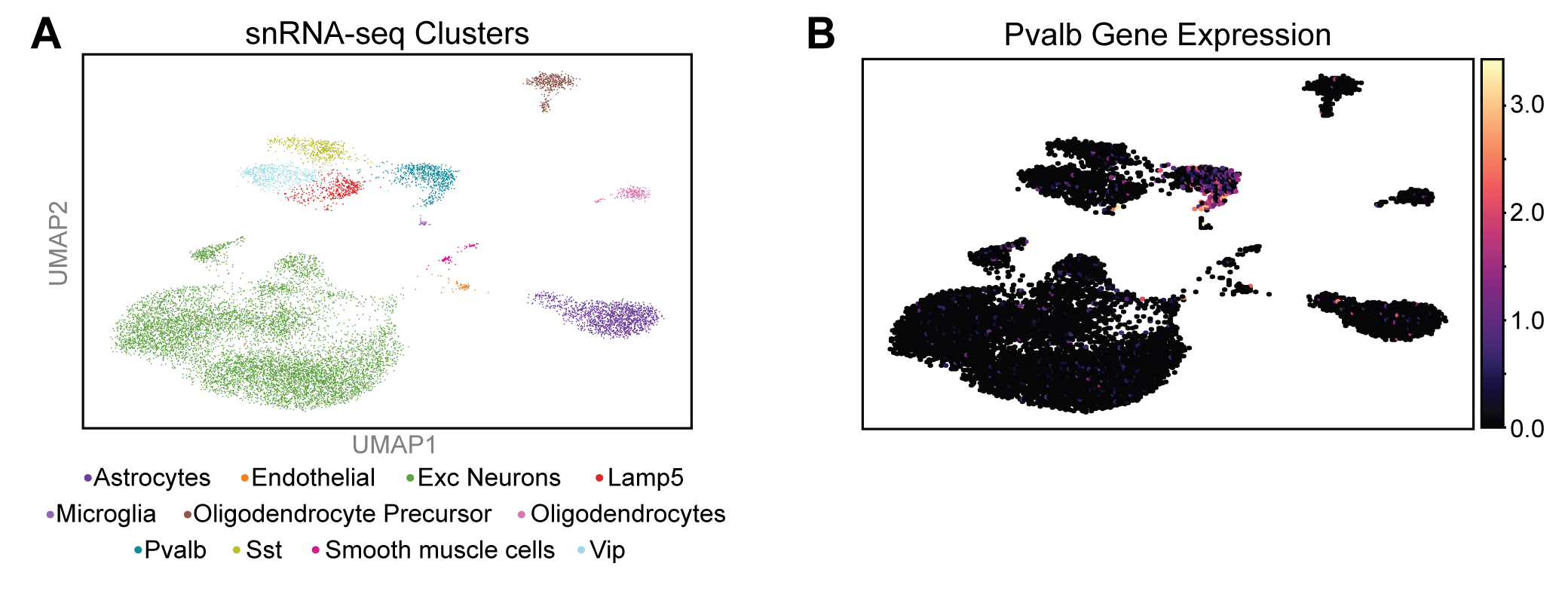

Supplement: Figure 2-2 — Additional properties of the mouse motor cortex snRNA-seq data. A. Cluster annotations per cell of the snRNA-seq data with UMAP embedding. B. Pvalb gene expression is highest within the Pvalb interneuron cluster. Download Figure 2-2, TIF file. [file ns-JN-RM-1634-20-s05.tif]

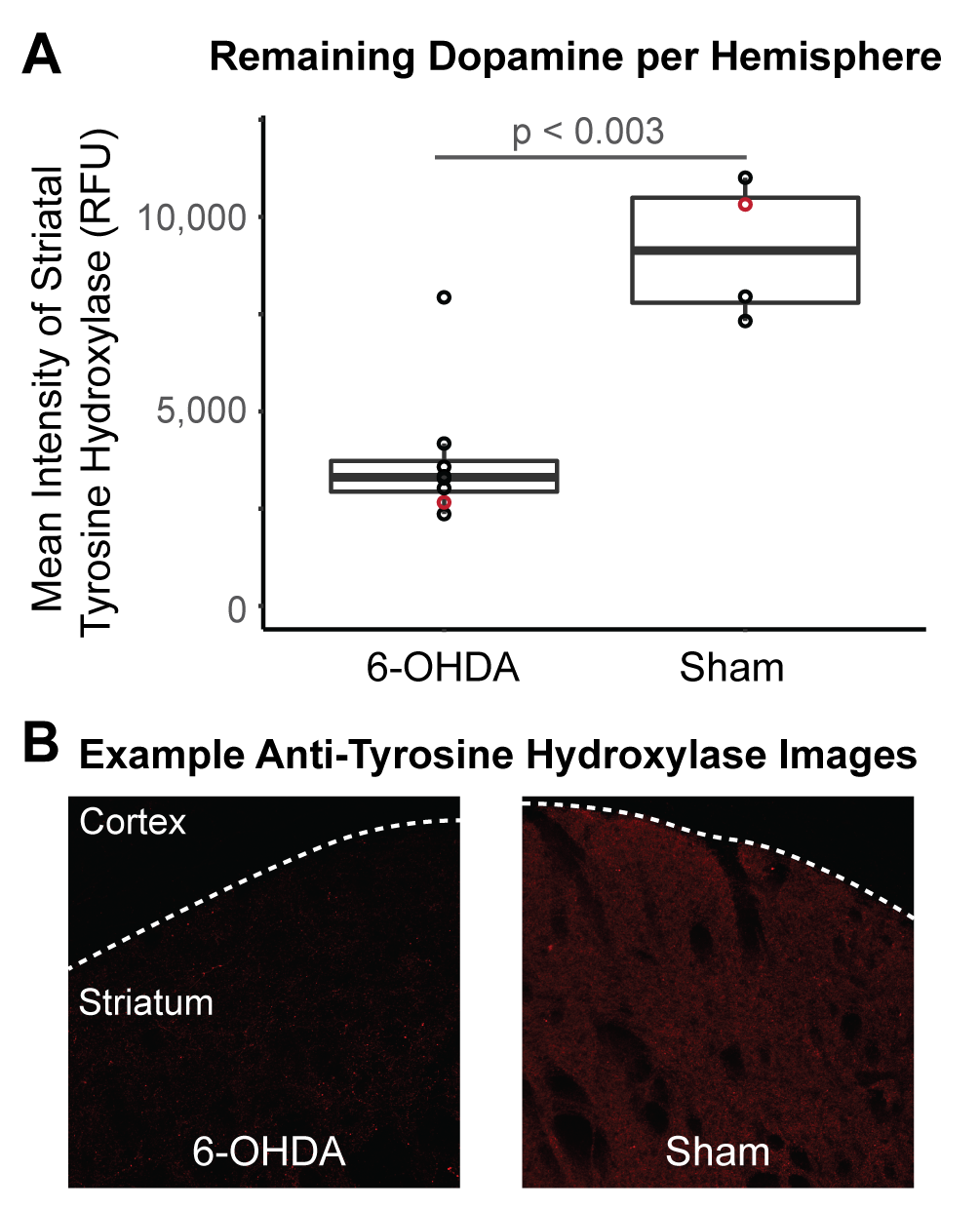

Supplement: Figure 4-1 — Quantification of dopamine depletion per hemisphere. A. Levels of striatal Tyrosine Hydroxylase for each hemisphere of the four 6-OHDA lesioned animals and 2 sham animals. The p value reflects a significant difference in populations by the standard t test. B. Examples of the quantified images, representing the two red points in A. Download Figure 4-1, TIF file. [file ns-JN-RM-1634-20-s06.tif]

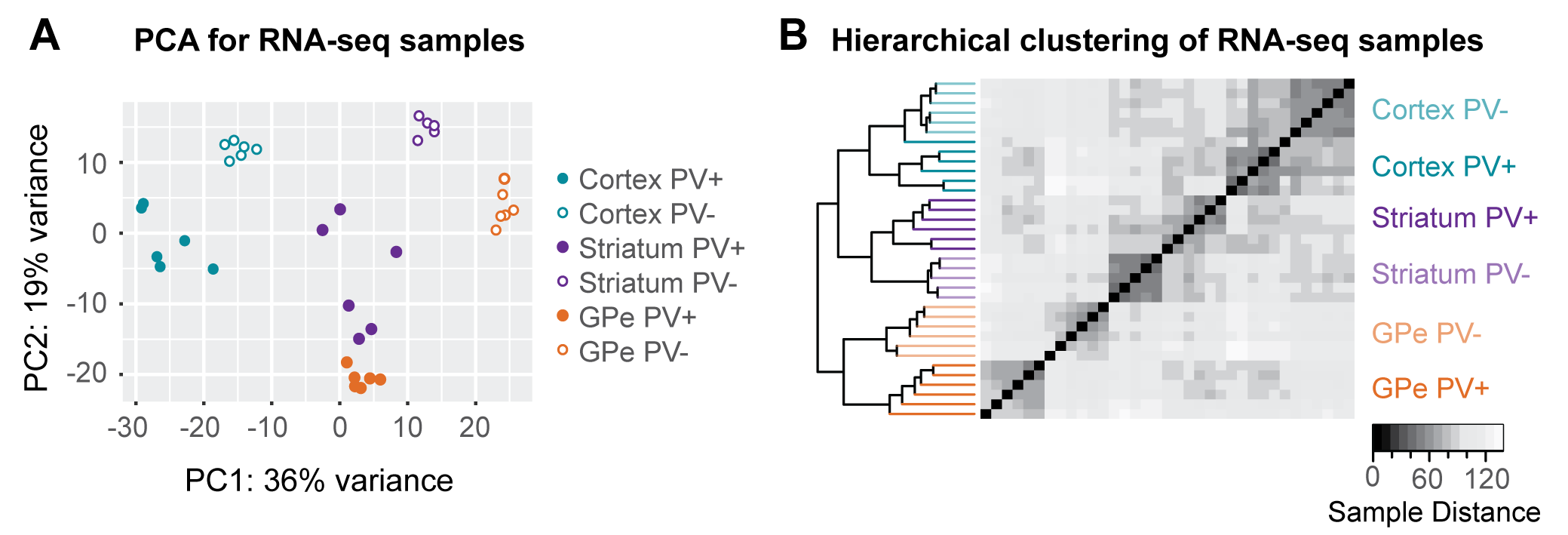

Supplement: Figure 4-4 — Example images of Hif2a staining in healthy and DD GPe tissue. Some instances of PV+Hif2a- cells are highlighted by white arrows. The scale bars measure 100 µm. Download Figure 4-4, TIF file. [file ns-JN-RM-1634-20-s07.tif]

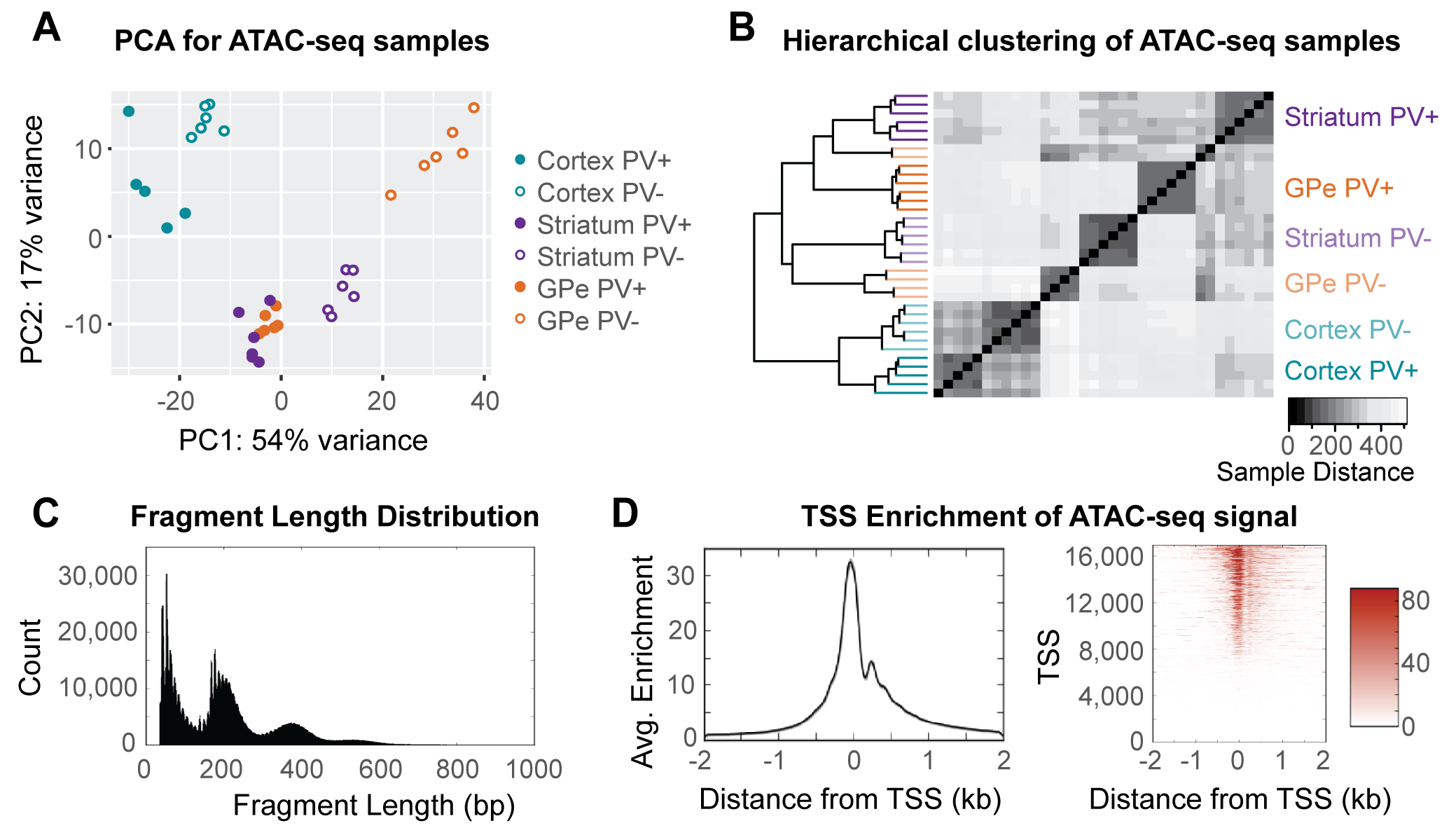

Supplement: Figure 5-1 — ATAC-seq data are high quality. A & B. ATAC-seq samples tend to cluster with other samples of the same tissue and cell type by PCA and hierarchical clustering of genome-wide open chromatin profiling. C. The data exhibit the characteristic periodicity in fragment length distributions of high quality ATAC-seq data, reflecting nucleosome positioning. The plot shown is a representative example from one GPe PV+ sample. D. ATAC-seq signal is enriched at transcription start sites (TSS), indicative of high signal-to-noise. The displayed data are a representative example from one GPe PV+ sample. Download Figure 5-1, TIF file. [file ns-JN-RM-1634-20-s08.tif]

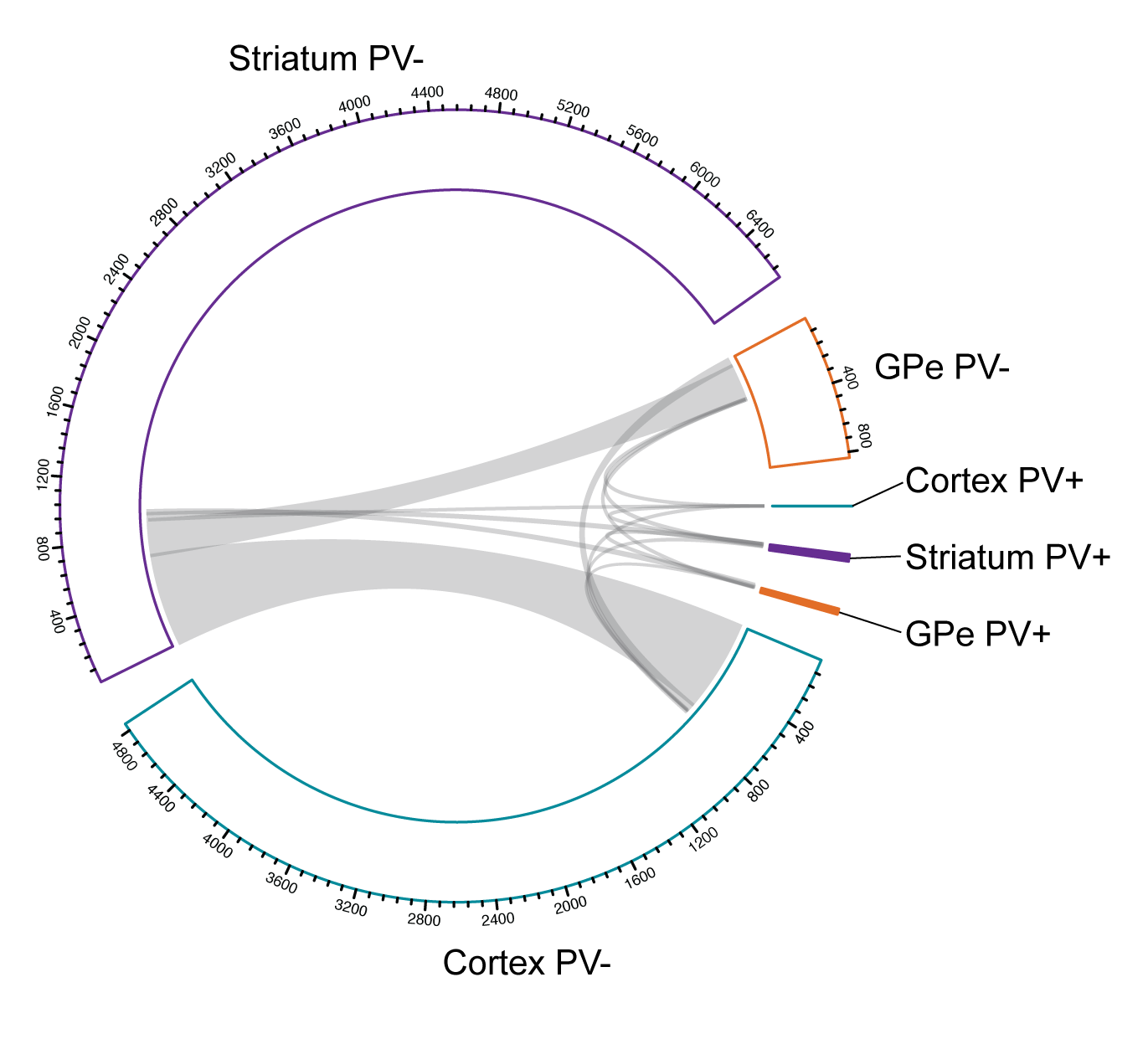

Supplement: Figure 5-3 — Overlap between DD-affected ATAC-seq peaks of different cell types. The width of each outer segment of the circle shows how many DD-affected peaks were recovered in that cell type. Bidirectional arcs between two cell types signify shared DD-affected peaks where the width of the connection represents the number of shared peaks. Plotted with circlize (Gu et al., 2014). Download Figure 5-3, TIF file. [file ns-JN-RM-1634-20-s09.tif]

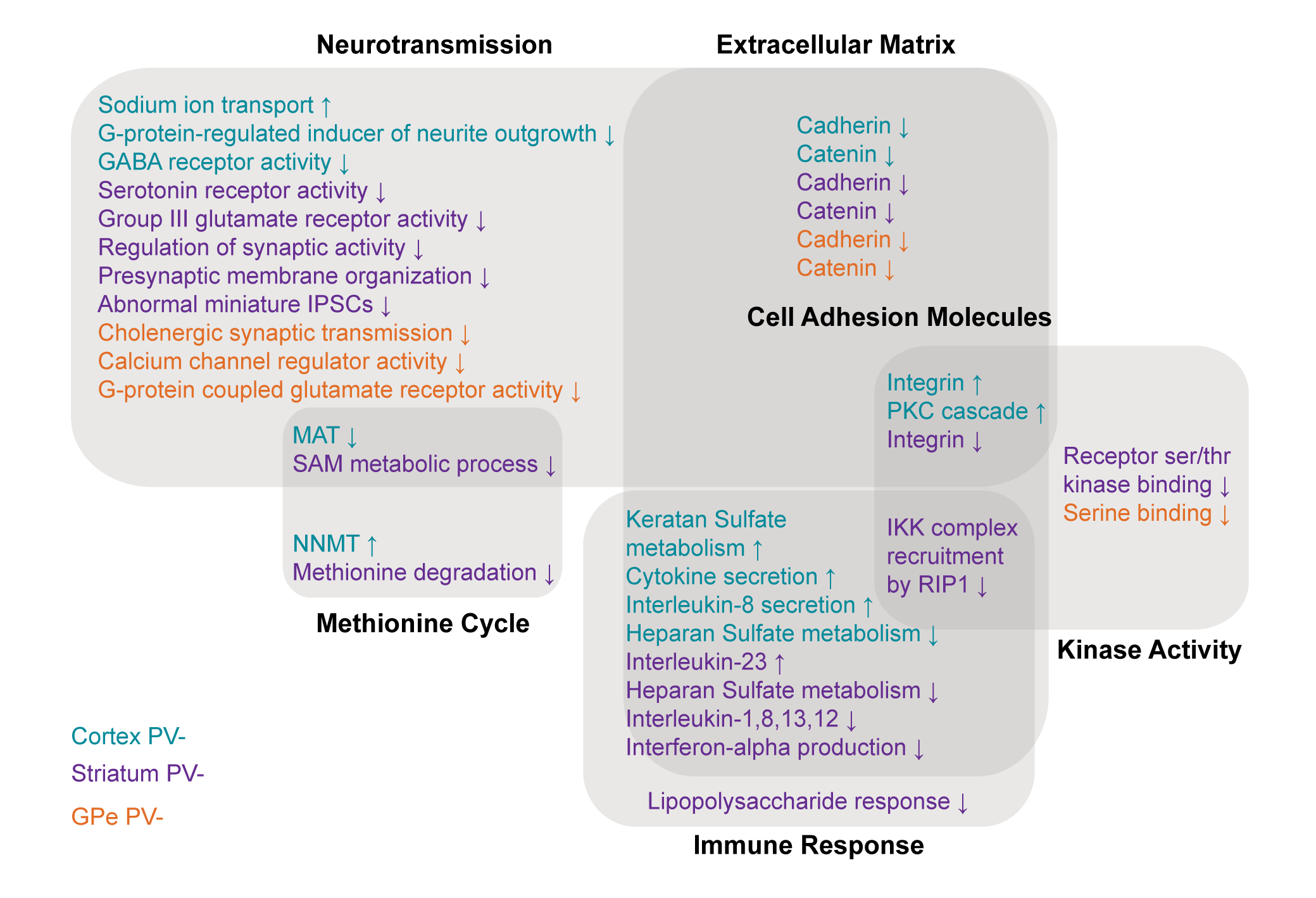

Supplement: Figure 5-4 — Summary of enriched annotations in DD-affected ATAC-seq peak sets. Enrichments were determined using GREAT for each differential set of peaks against a background of all peaks in that cell type. The arrow of a term indicates that it was enriched in DD-increasing peaks (up arrow) or DD-decreasing peaks (down arrow) and the color indicates the cell population. Download Figure 5-4, TIF file. [file ns-JN-RM-1634-20-s10.tif]

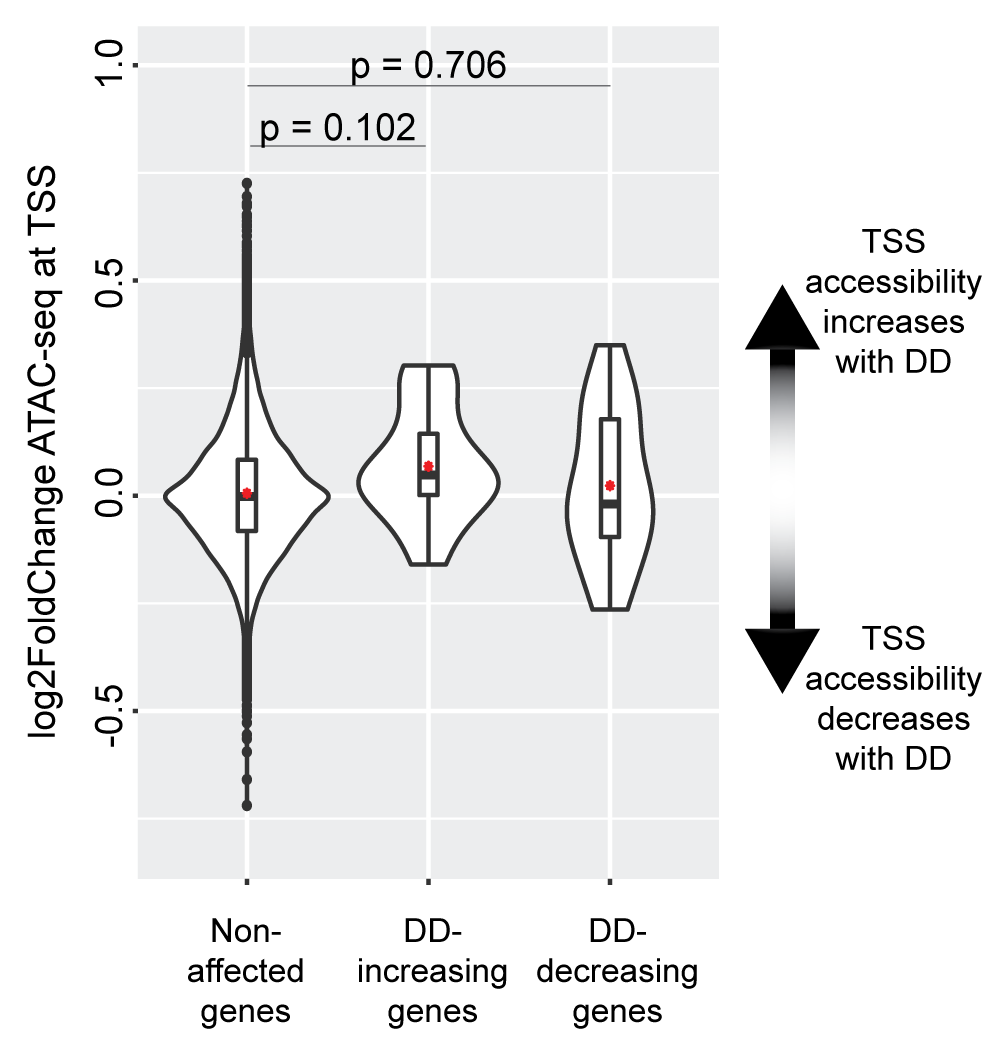

Supplement: Figure 5-6 — TSS accessibility of DD-affected genes in GPe PV+ neurons. The mean of each population is marked with the red star and the reported p values denote the significance of the difference in means by the two-tailed t test. Download Figure 5-6, TIF file. [file ns-JN-RM-1634-20-s11.tif]
